# Comprehensive Analysis of the Putative Substratome of FAM20C, the Master Serine Kinase of the Secretory Pathway

**DOI:** 10.3390/biom15111582

**Published:** 2025-11-11

**Authors:** Luca Cesaro, Francesca Noventa, Trinidad De Los Angeles Cordero, Barbara Molon, Valentina Bosello Travain, Maria Cristina Aspromonte, Mauro Salvi

**Affiliations:** 1 Department of Biomedical Sciences, University of Padova, 35131 Padova, Italy; 2 Department of Molecular Medicine, University of Padova, 35131 Padova, Italy

**Keywords:** protein phosphorylation, kinase consensus sequence, blood coagulation, complement system

## Abstract

FAM20C, previously known as Golgi casein kinase (GCK), is a serine/threonine kinase localized to the Golgi apparatus and classified within the acidophilic kinase family. Its phosphorylation motif is characterized by a glutamic acid residue at the +2 position relative to the target site. Before its molecular identity was established, analysis of a limited number of phosphosites in secreted proteins showed that around 70% matched the GCK consensus sequence, suggesting that GCK is the principal kinase for secreted proteins. Following the identification of GCK as FAM20C, the generation of FAM20C knockout cell lines and phosphoproteomic data confirmed its role: approximately 80% of serine/threonine phosphosites in the secretome of two different human cell lines were shown to depend on FAM20C. In this study, comparative analysis of in vitro phosphorylation datasets from a broad panel of recombinant Ser/Thr kinases confirmed that the FAM20C consensus sequence is distinct from those of other acidophilic kinases. Examination of experimentally identified human phosphosites within the secretory pathway revealed strong conservation of the FAM20C consensus, firmly establishing this enzyme as the master Ser kinase of the entire pathway. From this dataset, we defined the putative FAM20C substratome, comprising 443 phosphosites across 256 proteins, ~77% of which had not been previously linked to FAM20C. This represents the most extensive FAM20C substratome to date and a valuable resource for functional studies. Notably, enrichment analysis highlights strong links between FAM20C and major extracellular pathways, including collagen fibril organization, complement activation, and blood coagulation, underscoring an underappreciated role for this kinase in regulating hemostasis and innate immunity.

## 1. Introduction

The first phosphoprotein, casein, was identified in 1883 by Hammarsten [1], and, decades later (1953), Burnett and Kennedy demonstrated casein phosphorylation using rat liver extract, providing early evidence of kinase activity [2]. Casein kinase I and II were later discovered using casein as a substrate, but these enzymes were eventually renamed, since they lacked the correct subcellular localization and did not recognize the SxE motifs found in casein [3]. In 1972, a casein kinase activity was detected in the Golgi apparatus of lactating mammary glands, and was named GCK (Golgi casein kinase) [4]. The development of a synthetic peptide substrate corresponding to a phosphorylation site in bovine β-casein (β(28–40)), selectively phosphorylated by GCK but not by CK1 or CK2, identified GCK as the most promising candidate for mediating casein phosphorylation in vivo [5,6].

In 2010, analysis of a limited set of phosphoserine/threonine residues in proteins extracted from secreted proteins (86 Ser/Thr phosphosites detected in human plasma and 92 phosphosites detected in cerebrospinal fluid) revealed that the majority of these phosphosites (50–70%) shared a single kinase consensus motif, the Golgi casein kinase (GCK) consensus sequence (S/TxE) (where phosphorylated serine can substitute the glutamic at +2 position), leading to the hypothesis that GCK was the principal serine/threonine kinase responsible for phosphorylating secreted proteins [7]. A major breakthrough came in 2012 with the molecular identification of FAM20C as the authentic GCK [8,9]. This late identification was due to the fact that FAM20C is an atypical protein kinase. Unlike conventional eukaryotic protein kinases (ePKs) [10], from which it does not phylogenetically branch, FAM20C possesses only a subset of the canonical ePK catalytic domain motifs, defining its unique place in the kinome.

A further breakthrough came in 2015, when Dixon and collaborators used FAM20C knockout cell lines to demonstrate that approximately 80% of phosphosites in secreted proteins were FAM20C-dependent (direct or indirect substrates) [11], confirming earlier predictions based only on the kinase motif [7]. This discovery established FAM20C as the primary kinase responsible for the phosphorylation of secreted proteins, a notable finding given the complexity of the human kinome, which comprises over 500 serine/threonine kinases [12]. Since secreted proteins account for up to 20% of cellular transcripts, especially in secretory organs like the pancreas and liver [13] the dominance of a single kinase in this space is both unexpected and remarkable.

FAM20C-mediated phosphorylation influences the folding, trafficking, and function of extracellular proteins (for review, see [14]). Its physiological importance is highlighted by Raine syndrome, a rare, autosomal recessive disorder caused by FAM20C loss-of-function mutations, characterized by defective bone mineralization that can lead to early death in its most severe form [15]. The disease pathology underscores FAM20C’s essential function in phosphorylating key secreted proteins, such as members of the secretory calcium-binding phosphoprotein (SCPP) family, fibroblast growth factor 23 (FGF23), and small integrin-binding ligand N-linked glycoprotein (SIBLING) family [9], all of which regulate bone homeostasis.

FAM20C is a Golgi-resident kinase with a short cytoplasmic N-terminus, a single transmembrane domain, and a luminal catalytic domain. A signal peptide anchors it in the Golgi membrane [16]. Interestingly, FAM20C also associates with endoplasmic reticulum (ER) proteins [17] and recent studies confirm its ability to phosphorylate ER-localized targets, including redox regulators such as protein disulfide isomerase (PDI) and Ero1α, as well as regulators of calcium signaling, including histidine-rich calcium-binding protein (HRC), calsequestrin 2, and STIM [17,18,19,20]. These findings highlight an important role for FAM20C in maintaining the ER redox homeostasis [17] and regulating endoplasmic/sarcoplasmic reticulum calcium stores [21]. Although the ER is positioned earlier in the secretory pathway, it is topologically continuous with the Golgi lumen. This observation suggests that FAM20C-mediated phosphorylation may occur before protein trafficking or via retrograde transport or dynamic shuttling of the kinase or its substrates.

Collectively, these observations support a model in which FAM20C substrates span the full continuum of the secretory pathway, including the ER, Golgi apparatus, plasma membrane (domains in the extracellular space), and extracellular space [22]. Importantly, the prevailing consensus is that physiological phosphorylation predominantly takes place within the secretory pathway, intracellularly, before protein release. Although FAM20C itself can be secreted, the occurrence of phosphorylation events in the extracellular space remains under debate [23].

The in vivo activity and biological function of FAM20C are often dependent to its paralog, FAM20A [24]. FAM20A is a pseudokinase that lacks catalytic activity but serves as an essential allosteric activator. Together, they can form a stable heterotetrameric complex, which is considered a key functional form of the kinase within the secretory pathway [25]. However, this regulatory partnership is context dependent. FAM20A and FAM20C do not always share identical expression patterns across all tissues. This explains why the loss of FAM20A does not completely abolish all FAM20C function in the body. Nonetheless, the physiological importance of the FAM20A-FAM20C complex is clearly demonstrated by the fact that genetic ablation of FAM20A leads to Amelogenesis Imperfecta, that phenocopy mutations in FAM20C itself [26].

In this study, we present new evidence that FAM20C acts as the master serine kinase of the secretory pathway and provide a comprehensive analysis of its substrate repertoire and biological functions.

## 2. Materials and Methods

### 2.1. Data Collection

Experimentally identified phosphosites were obtained from the PhosphoSitePlus database [27]. The dataset was filtered to include only Ser or Thr phosphosites located on proteins annotated to the secretory pathway, cytosol, or nuclear matrix.

Validate FAM20C direct substrates used to generate the kinase consensus sequence were collected from the PhosphoSitePlus database [27] and from literature. We included both proteins and peptides that have been experimentally demonstrated to be directly phosphorylated by FAM20C, at least in vitro. To identify the putative FAM20C substratome, we queried the PhosphoSitePlus database [27] and selected all proteins annotated to the Golgi lumen, endoplasmic reticulum lumen, or extracellular matrix/region/space that contain at least one phosphorylated serine within the motif S-x-E. This initial dataset was then manually curated to refine the list of high-confidence potential substrates including only those localized within the secretory pathway (e.g., ER, and Golgi lumen). We excluded sites from proteins only cytoplasmically associated with the secretory pathway as well as phosphosites of membrane proteins not located in intraluminal or extracellular domains, based on UniProt annotations and Phobius-predicted topologies [28]. We also excluded phosphosites from proteins that, although released into the extracellular space, do not transit through the secretory pathway but are secreted via alternative mechanisms.

### 2.2. Analysis of Conserved Residues in Peptide Sequences

Amino acid conservation analysis of FAM20C direct substrate peptides (from positions −7 to +7 relative to the phosphorylation site), as well as phosphopeptides from various subcellular compartments, was performed using the Probability Logo Generator (pLogo), a motif visualization tool that represents the statistical significance of amino acid residues through scaled letter heights [29]. To generate a background dataset, random human peptides of the same length (positions −7 to +7) were extracted from the Swiss-Prot database using a custom BioPerl script and UNIX text processing commands. Non-redundant sequences were then randomized using the UNIX shuff command. Venn diagrams have been generated using the free tool Venn diagram generator (https://venndiagram.imageonline.co/).

### 2.3. Heatmap Generation

The heatmap was produced using the R package Superheat https://rlbarter.github.io/superheat/ version 1.0.0 (accessed on July 2025).

### 2.4. GO Annotation

Gene annotations, including subcellular components, biological functions, and BioPlanet pathways, were obtained using GeneCodis4 [30].

### 2.5. Sequence Retrieval, Alignment, and Filtering of Orthologues

Protein sequences of human complement and coagulation cascade members regulated by FAM20C were retrieved from the UniProt database [31] and used as query sequences. For each protein, a BLAST (Basic Local Alignment Search Tool) search [32] (version 2.13.0) was performed to identify orthologous sequences across a broad taxonomic range. To ensure evolutionary representation, orthologues were selected to cover diverse animal classes.

To meet the requirements of SplitsTree (v6.4.17) [33,34] for consensus tree and network construction, orthologues from a consistent set of species within each taxonomic class were retained. This strategy ensured both phylogenetic comparability and compatibility across all proteins included in downstream analyses.

Multiple sequence alignments (MSAs) were generated using MAFFT (v7.310-1) [35,36], employing the progressive G-INS-i algorithm [37]. Sub-alignments were then merged for each protein using the MERGE option in MAFFT. To improve alignment quality, MUSCLE (implemented in Jalview v2) [38] was used for further refinement. Manual curation was then conducted to ensure the accurate alignment of conserved SxE motif sites.

### 2.6. Phylogenetic Inference and Network Construction

Phylogenetic trees were inferred using W-IQ-TREE [39], with 1000 to 3000 ultrafast bootstrap replicates per protein to assess branch support. Maximum likelihood (ML) inference was applied, and the optimal substitution model for each protein was selected based on the Bayesian Information Criterion (BIC) (Figure S2).

Tree topology was evaluated using Robinson–Foulds (RF), clade, and Euclidean distance metrics (Figure S2) to assess consistency and structural congruence across proteins.

Phylogenetic trees were then used as input for SplitsTree (v6.4.17) [33,34], which generated a consensus tree, a consensus network, and a supernetwork. This network-based approach accounts for conflicting phylogenetic signals that may arise from evolutionary complexities, such as the influence of the SxE motif within the dataset.

### 2.7. Identification and In Silico Analysis of Pathogenic Variants in FAM20C Substrate Phosphorylation Motifs

To investigate the potential clinical impact of mutations affecting FAM20C-regulated phosphorylation motifs, we systematically screened all FAM20C substrates of Table S2 for missense variants classified as Pathogenic or Likely Pathogenic within conserved SxE motifs. Variant data were obtained from the ClinVar [40] and ClinGen [41] databases. Protein sequences were cross-referenced using UniProt accession numbers to confirm residue positions. Motif annotation was based on previously curated FAM20C substrate datasets.

## 3. Results

### 3.1. Is the FAM20C Kinase Consensus Sequence Sufficiently Distinctive to Serve as a Characteristic Signature of the Kinase?

FAM20C is an acidophilic kinase whose consensus phosphorylation motif differs notably from those of the well-characterized acidophilic kinases CK2 and CK1 [5]. Our previous work has shown that FAM20C’s unique preference for phosphorylating target sites containing an acidic residue specifically at the +2 position is not shared by other common acidophilic kinases, including PLK1, PLK2, PLK3, and GRK2 [42].

**Figure 1:**
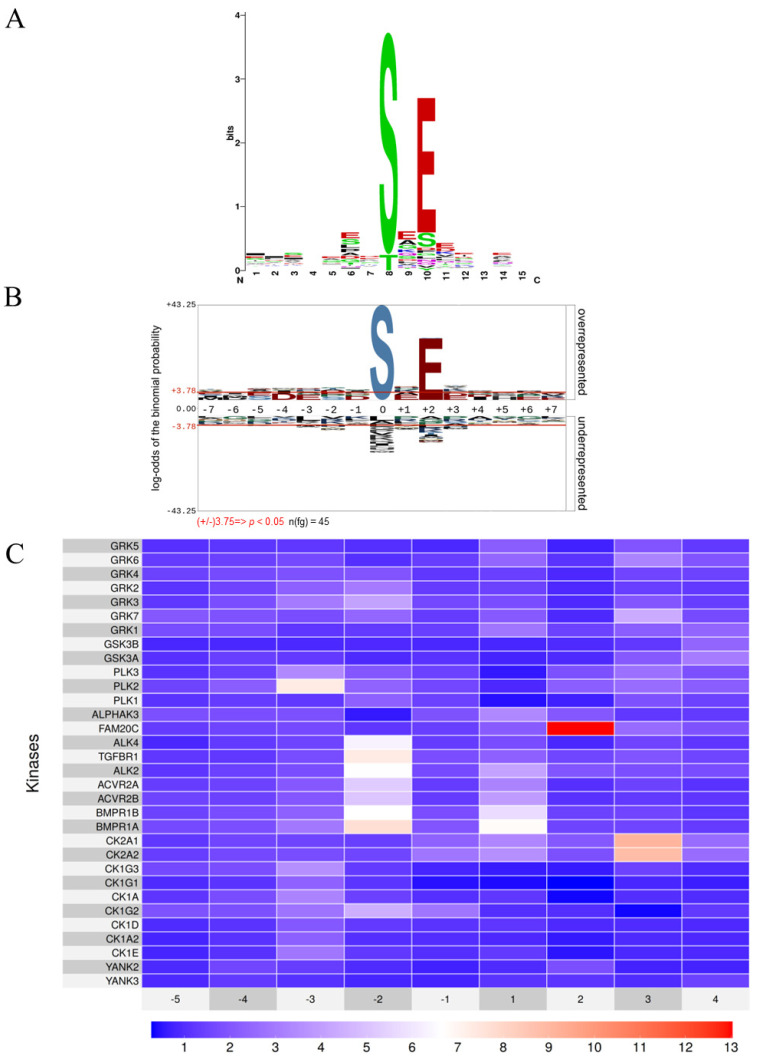
FAM20C kinase consensus sequence. (**A**,**B**). The primary sequences surrounding the FAM20C-generated phosphosites (from positions −7 to +7) from Table S1 were aligned using weblogo [43] (**A**), or pLogo [29] (**B**) with a background dataset consisting of random human peptides of the same length. (**C**) The recurrence of glutamic acid in the consensus sequences of acidophilic kinases was highlighted using a heatmap, as described in Materials and Methods. This heatmap was generated from data obtained through in vitro phosphorylation of a peptide library [12].

In this study, we present an updated and comprehensive compilation of all known phosphosites phosphorylated by FAM20C, whether identified through vitro assays or in cell-based studies, and whether on synthetic peptides or full-length proteins (Table S1). To analyze sequence motifs within these phosphosites, we utilized two motif visualization tools, WebLogo [43] and pLogo [29]: WebLogo (Figure 1A) displays amino acid conservation through sequence logos, while pLogo (Figure 1B) scales the height of each residue based on its statistical significance relative to a background distribution [29]. The height of each amino acid in the logos reflects its degree of conservation or statistical enrichment. Both representations highlight the strong conservation of glutamic acid residue at the +2 position (~78% of the phosphosites contain a glutamic acid residue at the +2 position.). Additionally, the WebLogo reveals a higher specificity for serine over threonine as the preferred phosphorylation site (Figure 1A) in agreement with previous published evidence [5,9] (Figure 1A). In the pLogo representation, residues are stacked vertically according to their statistical relevance, with the most significant residues positioned closest to the *x*-axis. Horizontal lines above and below the *x*-axis indicate Bonferroni-corrected significance thresholds. This analysis reveals that the presence of a glutamic acid residue at the +2 position is the only statistically significant feature, occurring in approximately 80% of all FAM20C substrate phosphosites (Figure 1B).

As above mentioned, in 2010 we identified the sequence S/TxE as the predominant motif in phosphorylated sequences of secreted proteins [7] suggesting GCK as the primary kinase responsible for phosphorylating the secretome. Prevision later confirmed by the molecular identification of GCK as FAM20C [9] and phosphoproteomic analysis of the secretome in FAM20C knockout cells [11].

Could there be other kinds of kinases with overlapping recognition motifs that have yet to be identified? A key breakthrough came from Cantley and colleagues, who profiled the substrate specificity of 303 Ser/Thr kinases, covering over 84% of those believed active in humans, using a synthetic peptide library [12]. Their study expanded the known repertoire of acidophilic kinases into eight distinct motif classes (YANK, CK1, CK2, TGFBR, FAM20C, PLK, GSK3, GRK), categorized mainly into those dependent on glutamic/aspartic acid residues (e.g., CK2, TGFBR, FAM20C, PLK) and those requiring phosphorylated residues (pS, pT, pY) as priming sites (e.g., YANK, CK1, GSK3, GRK) [12]. Using data from Cantley’s study [12], we examined the substrate sequence preferences of all acidophilic kinases, focusing on their dependency on a glutamic acid of AA surrounding the phosphorylation site. As demonstrated in the heatmap of Figure 1C, which illustrates the conservation of glutamic acid in the residues surrounding the target site, FAM20C is the only kinase among the 32 acidophilic kinases profiled that exhibits a strong preference for glutamic acid at the +2 position.

This distinct substrate preference highlights the unique nature of the FAM20C consensus sequence, distinguishing it from all other acidophilic kinases analyzed. Another aspect that should be taken into consideration is the subcellular localization of these acidophilic kinases. Notably, among the 32 acidophilic kinases identified in the Cantley study [12], FAM20C is the only one localized in the lumen of the Golgi apparatus and the only one that functions within the secretory pathway. In contrast, all other kinases exhibit cytoplasmic, nuclear, or plasma membrane (with the catalytic domains oriented toward the cytosol) localization.

Therefore, the high specificity of the FAM20C consensus motif, combined with its unique subcellular localization, provides an effective tool for predicting putative substrates through the analysis of phosphosite databases.

### 3.2. FAM20C Motif in the Secretory Pathway

**Figure 2:**
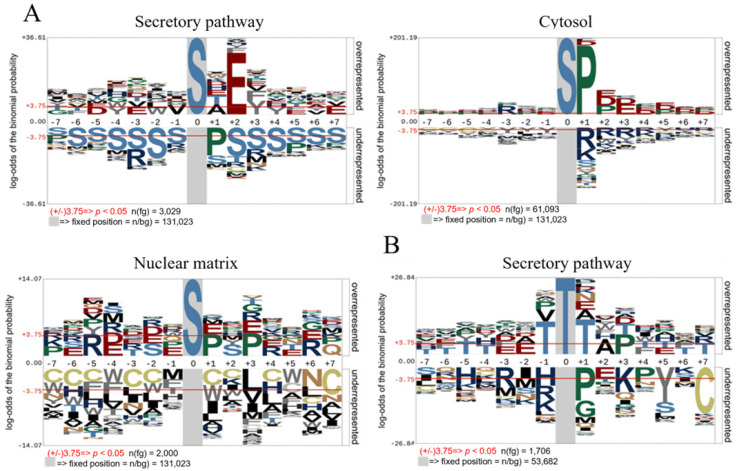
Analysis of the primary structure of human phosphosites in different subcellular localization. (**A**) pLogo of the primary structure of human Ser-phosphosites of proteins present in the secretory pathway, cytosol, or nuclear matrix, with a background dataset consisting of random human peptides of the same length. (**B**) pLogo of the primary structure of human Thr-phosphosites of proteins present in the secretory pathway, with a background dataset consisting of random human peptides of the same length.

To highlight the contribution of FAM20C across the entire secretory pathway, we curated human serine/threonine phosphorylation sites from the PhosphoSitePlus database (March 2024 release) [27], focusing on proteins localized to the extracellular space, Golgi lumen, and endoplasmic reticulum lumen. Notably, the majority of these phosphosites were identified through large-scale phosphoproteomic studies, and in most cases, the kinase responsible for their phosphorylation remains unknown. Sequence motifs surrounding phosphorylation sites (+7/−7 amino acids) were analyzed using pLogo [29], comparing residue conservation against a background database of random peptides. To further dissect FAM20C’s substrate specificity, we performed separate analyses on phosphorylated serine and phosphorylated threonine residues, based on previous evidence of FAM20C’s strong preference for serine over threonine. The pLogo analysis of P-Ser peptides from the secretory pathway, encompassing 3029 phosphopeptides, confirms that the SXE motif is the predominant consensus sequence (Figure 2A). Within this window, glutamic acid at the +2 position is the most conserved residue, supporting FAM20C’s role as the master kinase for serine phosphorylation of proteins in the secretory pathway. Conversely, the same analysis applied to P-Thr residues reveals that the TxE motif is underrepresented (Figure 2B), indicating a minimal role for FAM20C in threonine phosphorylation within this compartment.

It is important to note that PhosphoSitePlus assigns proteins to specific compartments based on gene ontology terms without individual verification, which may introduce background noise due to inaccurate or overlapping subcellular annotations. For example, some proteins detected outside the cell may not originate from the secretory pathway, while others associated with the endoplasmic reticulum or Golgi apparatus may only be linked to, rather than located within, their luminal spaces. Membrane proteins with cytosolic domains may also contribute to this noise. Nevertheless, when considered on a larger scale, these limitations do not substantially affect the conclusions of the analysis; rather, they reinforce the robustness of the observed trend, which clearly emerges above the background variability. To emphasize the predominance of the FAM20C consensus sequence in the phosphorylation of serine residues in the secretory pathway, we conducted a comparative analysis on conservation of residues surrounding phospho-serine collected from other subcellular compartments. These analyses show completely different conservations patterns (Figure 2A). In cytosolic proteins, a proline residue at the +1 position is the most conserved, consistent with phosphorylation by proline-directed kinases such as those in the MAPK cascade, which play a dominant role in cytosolic serine phosphorylation. Additionally, an enrichment of acidic residues downstream of the phosphorylation site is observed, characteristic of acidophilic kinases like CK2 [44] as well as the presence of an arginine at the −3 position, a key determinant for recognition by basophilic kinases such as Akt and PKB [45]. For phospho-serine in proteins localized to the nuclear matrix, a still different conservation pattern was detected compared to the cytosolic phosphosites with a minor general conservation, even if some features highlighted in the cytosol, such as the Proline in +1 and the arginine in +3, are still present (Figure 2A).

### 3.3. FAM20C Putative Substratome

In view of the latest findings (namely, the presence of a distinctive FAM20C consensus sequence, its unique subcellular localization among acidophilic kinases, and its central role in serine phosphorylation within the secretory pathway), we extracted from the secretory pathway phosphoproteome all phosphosites matching the canonical pSxE motif.

To assemble the FAM20C potential substratome, we manually curated phosphosites to retain only those localized within the lumen of the secretory pathway, including the extracellular space. Phosphosites from proteins associated solely with cytoplasmic regions of the secretory pathway and from membrane proteins not residing in intraluminal or extracellular domains were excluded, according to UniProt annotations and Phobius-predicted topologies. We also excluded phosphosites from proteins that, although released into the extracellular space, do not transit through the secretory pathway but are secreted via alternative mechanisms.

This analysis collected 443 phosphosites across 256 proteins, which are comprehensively listed in Table S2 and represent the putative full FAM20C substratome. It is important to note, however, that while this may represent the most comprehensive FAM20C substratome available to date, it likely remains incomplete. It does not include substrates whose phosphorylation sites have not yet been identified or are absent in PhosphositePlus database, nor does it capture non-canonical FAM20C substrates that rely on alternative or less common consensus sequences [46,47].

To distinguish between previously identified and newly predicted components of the putative FAM20C substratome, we compiled Tables S3 and S4. Table S3 lists known human protein direct FAM20C substrates, including proteins for which the specific phosphorylation site(s) remain(s) unidentified. Table S4 reports human phosphosites whose phosphorylation is reduced in FAM20C knockout or knockdown cells, match the FAM20C consensus sequence (SXE) and localize within the secretory pathway.

**Figure 3:**
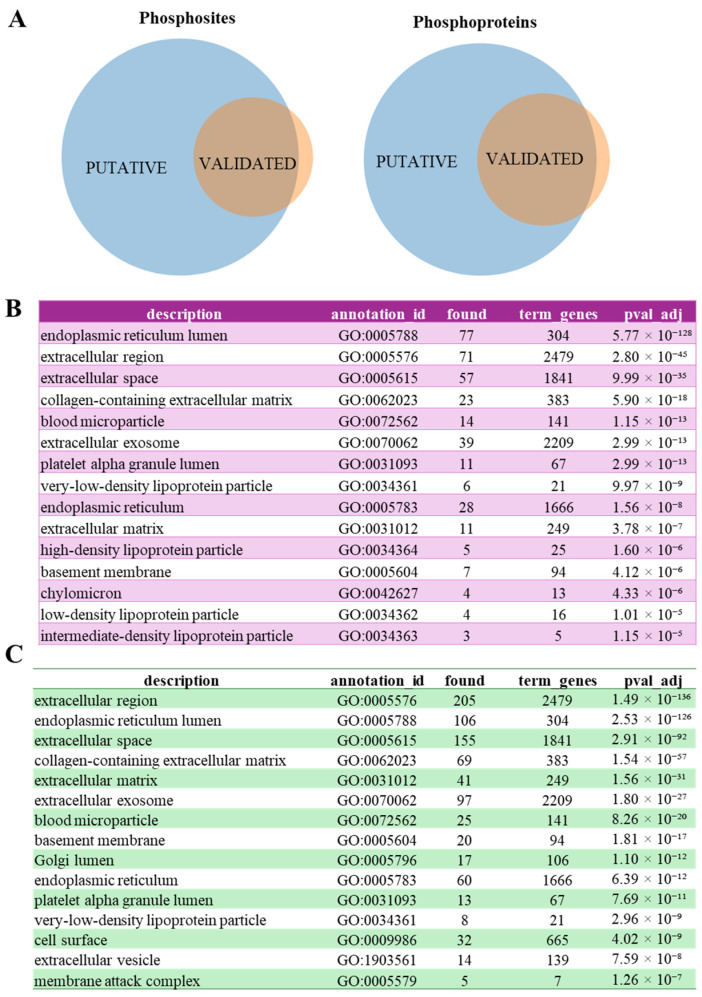
Validated vs. putative FAM20C substratome: Cell component (CC) annotation of FAM20C substrates. (**A**) Venn diagram highlighting the overlapping between phosphosites or protein in validated and putative FAM20C substratome. (**B**,**C**) Cell component (CC) annotation of validated FAM20C substratome (**B**) or the putative FAM20C substratome (**C**). Annotations were performed using Genecodis [30].

In Table S2 phosphosites already described as FAM20C substrates, either as direct substrates (listed in Table S1) or phosphosites whose phosphorylation is influenced by FAM20C modulation (listed in Table S4), are highlighted in red. Notably, 342 out of 443 (about 77%) of the listed phosphosites have not yet been identified as FAM20C substrates (Figure 3A).

Similarly, proteins previously confirmed as FAM20C substrates (Tables S3 and S4) are marked in red. This analysis reveals that 178 out of 256 (about 70%) of the listed proteins have not yet been identified as FAM20C substrates (Figure 3A).

These analyses indicate that our current understanding of FAM20C is still far from complete.

To assess the potential functional relevance of our putative FAM20C substratome, we analyzed its gene ontology (GO) annotations and compared them with a "validated substratome" comprising all proteins that are either confirmed direct substrates (Table S3) or likely direct substrates (i.e., proteins that show decreased phosphorylation upon FAM20C modulation, contain the FAM20C consensus motif, and are localized within the secretory pathway (Table S4).

The validated substratome indicates that the majority of FAM20C substrates are either endoplasmic reticulum (ER)-resident proteins or extracellular (Figure 3B). The substantial number of ER-localized substrates is consistent with previous immunoprecipitation-based interactome data, which revealed a greater number of FAM20C interactors in the ER compared to the Golgi apparatus [17]. A comparison of the subcellular localization profiles between the validated and putative substratomes (Figure 3B vs. Figure 3C) reveals a broadly similar distribution. The most notable changes in the putative substratome are a marked increase in extracellular proteins and the emergence of the Golgi lumen and cell surface among the top enriched compartments. This indicates that our predictive analysis not only expands the list of extracellular substrates but also identifies new potential candidates in the entire secretory pathway.

**Figure 4:**
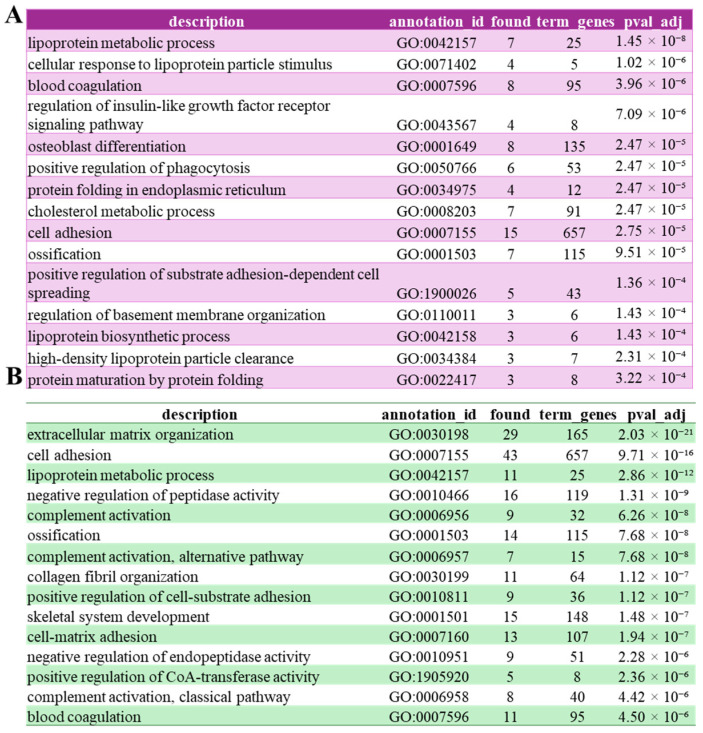
Biological Function (BP) annotation of FAM20C validated and putative substrates. Cell component (CC) annotation of validated FAM20C substratome (**A**) or the putative FAM20C substratome (**B**). Annotations were performed using Genecodis [30].

To gain insights into potential novel roles of FAM20C, we compared the functional annotations of the validated FAM20C substratome with those of our putative substratome. Gene ontology enrichment analysis of the validated FAM20C substratome revealed a strong association with biological processes related to lipid and lipoprotein metabolism. In particular, terms such as lipoprotein metabolic process, cholesterol metabolic process, and high-density lipoprotein particle clearance highlight a potential role of FAM20C in regulating lipid homeostasis. Processes including blood coagulation, ossification, and osteoblast differentiation are consistent with the known involvement of FAM20C in biomineralization and extracellular protein phosphorylation. Additional enriched terms, such as protein folding in the endoplasmic reticulum and protein maturation by protein folding, point to a link between FAM20C activity and secretory protein quality control and processing. GO Biological functions analysis of the putative substratome revealed an enrichment that expands the biological scope of FAM20C-associated processes. In addition to the pathways identified in the validated substratome, this extended dataset shows a strong overrepresentation of terms related to extracellular matrix organization, collagen fibril organization, cell adhesion, and cell–matrix interactions, highlighting an even broader involvement of FAM20C in extracellular structural dynamics. Moreover, the enrichment of complement activation (classical and alternative pathways) and negative regulation of peptidase and endopeptidase activity suggests a possible role for FAM20C in modulating immune and proteolytic processes within the extracellular environment. The persistence of terms such as ossification, skeletal system development, and blood coagulation further reinforces the functional link between FAM20C and key secretory and extracellular pathways (Figure 4B). Regarding collagen fibril organization, a previous study in which FAM20C was selectively inactivated in Type I collagen-expressing cells demonstrated that collagen fibrils in FAM20C-deficient bone were disorganized and abnormally thick [48]. These findings support our analysis suggesting a direct or indirect role for FAM20C in regulating collagen fibril organization.

**Figure 5:**
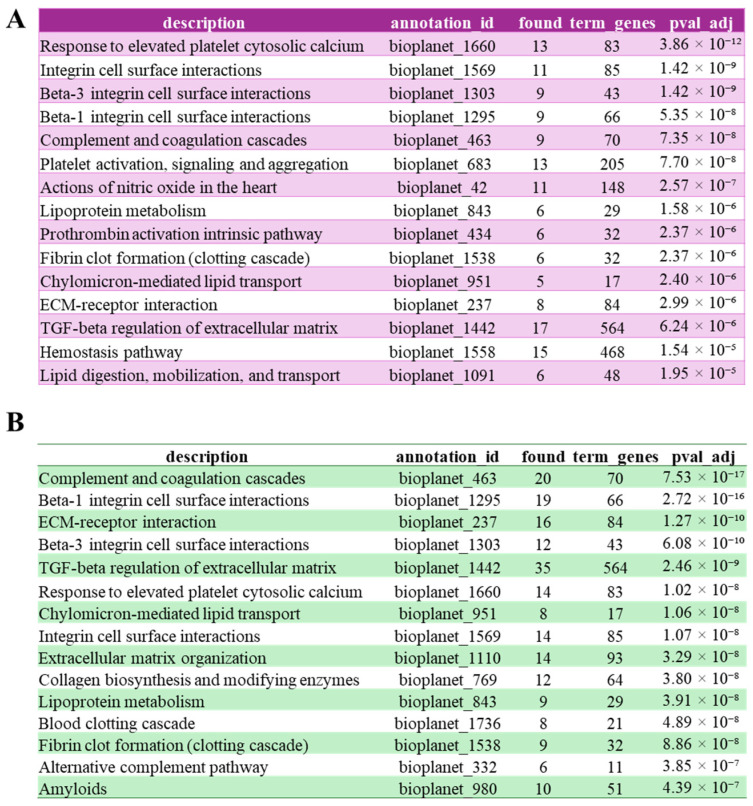
Bioplanet annotation of FAM20C validated and putative substrates. Cell component (CC) annotation of validated FAM20C substratome (**A**) or the putative FAM20C substratome (**B**). Annotations were performed using Genecodis [30].

BioPlanet pathway enrichment (Figure 5) confirmed that FAM20C substrates are predominantly involved in extracellular and secretory functions. In the validated substratome, pathways such as integrin-mediated cell surface interactions and ECM–receptor interaction, were most prominently represented. In contrast, within the expanded putative substratome, the complement and coagulation pathways, already detected in the validated substratome but ranked only fifth, emerged among the top enriched categories, suggesting that FAM20C may influence these processes more extensively than previously appreciated. This dataset also revealed additional enrichment for TGF-β regulation of the extracellular matrix and other fibrotic processes, together with collagen biosynthesis and modifying enzymes, extracellular matrix organization, and amyloid-related processes. These results point to an extended regulatory role for FAM20C in extracellular protein remodeling and homeostasis. Overall, these findings reinforce the link between FAM20C-dependent phosphorylation and the coordinated regulation of matrix dynamics, coagulation, and immune pathways in the secretory environment. Although the involvement of FAM20C in blood coagulation has been previously suggested, particularly through the phosphorylation of von Willebrand factor (VWF), which enhances its adhesiveness to platelets in vitro [49], our analysis indicates that this role may be far more extensive than previously appreciated. Interestingly, a mild coagulation defect was incidentally observed during surgery in a patient with Raine syndrome [50], the autosomal recessive disorder caused by FAM20C mutations [15], further supporting a possible functional link between FAM20C and coagulation pathways. Regarding the complement components, while only a few complement factors have been previously validated as FAM20C substrates, our analysis reveals a substantial expansion in the number of complement components identified as potential targets of the kinase, spanning both the classical and alternative pathways. Figure S1 visually illustrates the differences between the validated and putative substratome within the coagulation and complement pathways, with FAM20C identified substrates highlighted in red.

### 3.4. Phylogenetic Structure and Network Signals of SxE-Containing Proteins in the Complement and Coagulation Cascades

To better understand the evolutionary pressures and functional conservation underlying FAM20C-regulated phosphorylation events, we performed a phylogenetic analysis of SxE-containing proteins in the complement and coagulation cascades (Figure 5). Our findings uncover a previously underappreciated role for FAM20C in modulating coagulation and complement system pathways. To investigate the evolutionary history of FAM20C-regulated proteins involved in these pathways, we analyzed the FAM20C putative substrates in the complement and coagulation cascades across mammals, birds, and amphibians. Multiple sequence alignments revealed a high degree of conservation for the SxE motif, both within and across taxonomic classes, supporting its broad evolutionary stability.

We constructed a phylogenetic tree for each protein, followed by a consensus tree (Figure S2) rooted with *Pleurodeles waltl*. Despite aligning different proteins, the consensus tree recapitulated known species phylogeny: major clades (e.g., artiodactyls, carnivores, proboscideans) showed strong bootstrap support (≥95%). This indicates that the evolutionary signal of host species dominates, and proteins cluster by taxonomy rather than function.

A consensus network (Figure S2B) highlighted regions of topological incongruence, especially involving *Homo sapiens*, *Mus musculus*, and *Loxodonta africana*. These nodes exhibited conflicting placements across individual protein trees, as evidenced by thick reticulated edges with low support values (e.g., 68.42%, 36.8%, 31.57%).

Notably, *Homo sapiens*, *Mus musculus*, and *Equus caballus* occupied ambiguous positions in the supernetwork (Figure S2C), which displayed a central star-like topology. These species connected directly to the central node, suggesting hard polytomies and unresolved evolutionary relationships. In contrast, other taxa (e.g., cetaceans, ungulates, birds) maintained stable groupings with minimal reticulation.

Proteins contributing most to network instability included vWF, TFPI, and Factor VIII, particularly in humans and rodents. These showed inconsistent placements across trees, hinting at possible divergent evolutionary trajectories.

This evolutionary framework positions FAM20C not just as a kinase, but as a conserved regulator maintaining essential phosphorylation events that vertebrate coagulation and complement systems cannot functionally compromise, even as other aspects of these pathways adapt to species-specific needs.

### 3.5. Pathogenic Variants Affecting FAM20C Substrates Phosphorylation Motifs in Human Diseases

Given the critical role of FAM20C in biomineralization and the phosphorylation of secretory proteins, mutations in the *FAM20C* gene and dysregulation of its kinase activity have been linked to several diseases, including Raine Syndrome (RS) [15], and other pathologies [51,52,53]. Recent studies have highlighted FAM20C involvement in tumorigenesis, suggesting its potential as both a biomarker and a therapeutic target across various cancers [53]. Importantly, FAM20C role in disease may be indirect, arising from dysregulation of signaling pathways involving its phosphorylated substrates. To explore this possible correlation, we screened the comprehensive FAM20C substratome for missense Pathogenic and Likely Pathogenic missense variants occurring in conserved SxE motifs (missense variants affecting the serine residue within the **S**xE motif and missense variants occurring at the +2 position relative to the phosphorylated serine-Sx**E**) using data collected in ClinVar [40] and ClinGen [41] resources. Most of the variants are currently of uncertain significance and require further investigation to clarify their pathological roles.

A variant in the gene *TTR* (encoding transthyretin, a homotetrameric protein whose function is to transport the thyroid hormone thyroxine and the retinol-binding protein bound to retinol, UniProt ID: P02766) (p.Ser72Pro) (associated with Amyloidosis, hereditary systemic 1-AMYLD1) affects a phosphorylated serine located in a flexible linker on the protein surface. Notably also two variants of the glutamic in +2 of the same protein (p.Glu74Ala and p.Glu74Gly) are classified as pathogenic/likely pathogenic and associated with AMYLD1. Two other *TTR* variants, p.Glu74Lys and p.Glu74Gln, are classified as Likely Pathogenic and Pathogenic in two independent studies and associated with AMYLD1, while a third study reports it as a Variant of Uncertain Significance.

We identified a variant in *PCSK9* (UniProt ID: Q8NBP7), p.Ser47Cys (NM_174936.4: c.140C>G), reported in patients with familial hypercholesterolemia (OMIM #603776). PCSK9 regulates cholesterol levels by promoting the degradation of LDL receptors thereby reducing the clearance of LDL cholesterol from the bloodstream. The role of PCSK9 phosphorylation by FAM20C have been investigated in a previous paper [54]. In particular the FAM20C-dependent phosphorylation of PCSK9 at Serine 47, 666, 668, and 688 enhances its activity whereas substitution of these residues with alanine significantly reduces its function [54]. Based on these findings, the p.Ser47Cys variant would be expected to diminish PCSK9 activity and thereby lower LDL cholesterol levels. However, its association with hypercholesterolemia suggests that the functional consequences of this mutation are more complex than anticipated, potentially involving structural, regulatory, or compensatory mechanisms beyond the loss of phosphorylation alone.

Finally, a Likely Pathogenic variant, p.Ser1517Arg, was found in *VWF* (encoding von Willebrand factor), and has been reported in a patient with von Willebrand disease type 2 (OMIM #613554), underscoring the potential role of FAM20C in the coagulation pathway.

## 4. Discussion

Our results provide a comprehensive and updated view of the FAM20C kinase consensus motif and its biological relevance within the secretory pathway, highlighting both the specificity and potential functional implications of this kinase’s substrate selectivity.

We confirmed that FAM20C displays a unique consensus motif (SxE), characterized by a strict requirement for a glutamic acid at the +2 position and a strong preference for serine over threonine as the phosphorylation site. This specificity clearly distinguishes FAM20C from all other acidophilic kinases identified to date by the Cantley study [11] (Figure 1B). Moreover, its exclusive localization to the Golgi apparatus, and consequently to the secretory pathway, makes FAM20C the only acidophilic kinase known to function within this subcellular environment to date. Our pLogo analysis of over 3000 phosphoserine peptides from secretory pathway proteins revealed a clear dominance of the SxE motif, indicating that FAM20C is likely the master serine kinase in this cellular compartment. The lack of conservation of the FAM20C motif in phosphothreonine peptides suggests a minimal contribution of FAM20C to threonine phosphorylation. Importantly, when comparing secretory phosphosites with those from the cytosol and nucleus, the conservation patterns differ significantly, confirming that the FAM20C motif is uniquely enriched in the secretory pathway.

By filtering all secretory phosphosites matching the canonical pSxE motif, we compiled a putative FAM20C substratome that includes 443 sites across 256 proteins. Notably, approximately 77% of these phosphosites and 70% of the corresponding proteins have not been previously associated with FAM20C, suggesting the significant underestimation of this kinase’s full functional role. These phosphosites are found in proteins distributed throughout the entire secretory pathway. While the majority correspond to classical secreted proteins located in the extracellular space, supporting a primary role of this kinase in the regulation of the extracellular milieu, a significant proportion of substrates are resident proteins of the ER and a few are localized in the Golgi apparatus. Of particular interest, two identified substrates are localized to the perinuclear space, a compartment topologically continuous with the ER, whose biological significance has thus far been largely overlooked. Gene Ontology analysis of the putative substratome further validated known FAM20C roles in extracellular matrix organization and ossification, but also revealed previously underappreciated associations with collagen fibril organization, complement activation and blood coagulation pathways. The enrichment of collagen-related proteins among the predicted substrates strengthens the hypothesis that FAM20C contributes to extracellular matrix assembly. Similarly, the identification of multiple putative FAM20C substrates involved in coagulation and complement pathways points to a broader role for FAM20C in hemostasis and immunity. Together, these findings advance our understanding of FAM20C biological function. The distinctive sequence motif, unique subcellular localization, and expanded substratome emphasize its critical role in orchestrating phosphorylation events across the secretory pathway.

Our integrated phylogenetic and network-based analysis on FAM20C substrates of coagulation and complement pathways reveals that the SxE motif is broadly conserved across diverse vertebrate lineages, reinforcing its likely functional importance. The clustering of species according to taxonomy, rather than protein function, indicates a strong signal of vertical inheritance for most proteins. However, the presence of phylogenetic conflicts, especially involving *Homo sapiens* and *Mus musculus*, suggests that certain proteins may have undergone lineage-specific adaptations. This is consistent with the idea that some immune or coagulation factors have experienced functional divergence, gene duplication, or accelerated evolution, particularly in mammals.

Previous studies support the co-evolution of proteins like Factor VIII and vWF under selective pressures [55,56], which could explain their unstable phylogenetic signals. Furthermore, the dense reticulation observed in networks may reflect heterogeneity in evolutionary rates, especially for proteins with specialized functions in humans or rodents.

Comparative genomics also reveals that genes involved in immune and reproductive functions often evolve rapidly in mammals, while core metabolic pathways remain conserved. Our data align with these findings: while most species exhibit stable evolutionary signals for SxE-containing proteins, a subset, especially in primates and rodents, shows evidence of functional innovation or motif divergence.

In sum, while the SxE motif is conserved, the evolutionary history of the proteins that bear it is not uniformly stable. Instead, it reflects a balance between structural conservation and functional divergence, shaped by lineage-specific pressures and biological demands.

This evolutionary perspective is further supported by our identification of pathogenic or likely pathogenic missense variants occurring within SxE motifs of four FAM20C substrate genes. These mutations are rare across the full set the putative substratome, underscoring the strong selective constraint acting on these motifs. These findings suggest that alterations at conserved SxE sites are not only evolutionarily constrained, but also clinically significant, particularly in proteins with central roles in coagulation and lipid metabolism.

Taken together, our data reveal a dual aspect of SxE motif conservation: it reflects a deep evolutionary history preserved across vertebrates yet also represents a functionally sensitive site where rare mutations can have profound structural and pathological consequences. The overlap between regions of phylogenetic instability and the occurrence of clinically relevant variants in humans and rodents may point to ongoing evolutionary innovation, possibly driven by immune or metabolic pressures unique to these lineages.

## 5. Conclusions

We demonstrate that the SxE motif is a highly specific signature of FAM20C. This signature is distinct from those of all other acidophilic kinases. Together with its exclusive localization to the Golgi apparatus, this specificity establishes FAM20C as the dominant kinase responsible for serine phosphorylation in the secretory pathway.

Our comprehensive analysis of the putative FAM20C substratome reveals that our current understanding of the functional role of FAM20C was underestimated. We have identified a multitude of novel putative substrates, substantially expanding the known FAM20C substratome and implicating this kinase in a broader range of biological processes. It should be emphasized that, although all these phosphosites have been experimentally identified mainly in large-scale phosphoproteomic studies, their direct phosphorylation by FAM20C has not been verified. While the presented evidence strongly suggests that FAM20C is potentially involved in the phosphorylation of these sites, direct experimental validation for each one is required.

Another critical consideration is that, although these sites are known to be phosphorylated, quantitative information regarding the extent of their modification and, more importantly, their functional relevance, is lacking. The latter is arguably the most crucial aspect, and future studies aimed at determining which of these phosphosites are most critical in regulating protein function will provide new insights into the real impact of FAM20C in the secretory pathway.

## Data Availability

The data that support the findings are available in Supplementary Materials.

## Supplementary Materials

The following supporting information can be downloaded at: https://www.mdpi.com/article/10.3390/biom15111582/s1.

## References

[1] Hammarsten O. (1883). Zur Frage, Ob Das Caseïn Ein Einheitlicher Stoff Sei. Biol. Chem..

[2] Burnett G., Kennedy E.P. (1954). The Enzymatic Phosphorylation of Proteins. J. Biol. Chem..

[3] Venerando A., Ruzzene M., Pinna L.A. (2014). Casein Kinase: The Triple Meaning of a Misnomer. Biochem. J..

[4] Ew B., Hm F., Jj B. (1972). Phosphorylation of Casein. Role of the Golgi Apparatus. J. Biol. Chem..

[5] Lasa-Benito M., Marin O., Meggio F., Pinna L.A. (1996). Golgi Apparatus Mammary Gland Casein Kinase: Monitoring by a Specific Peptide Substrate and Definition of Specificity Determinants. FEBS Lett..

[6] Lasa M., Marin O., Pinna L.A. (1997). Rat Liver Golgi Apparatus Contains a Protein Kinase Similar to the Casein Kinase of Lactating Mammary Gland. Eur. J. Biochem..

[7] Salvi M., Cesaro L., Tibaldi E., Pinna L.A. (2010). Motif Analysis of Phosphosites Discloses a Potential Prominent Role of the Golgi Casein Kinase (GCK) in the Generation of Human Plasma Phospho-Proteome. J. Proteome Res..

[8] Ishikawa H.O., Xu A., Ogura E., Manning G., Irvine K.D. (2012). The Raine Syndrome Protein FAM20C Is a Golgi Kinase That Phosphorylates Bio-Mineralization Proteins. PLoS ONE.

[9] Tagliabracci V.S., Engel J.L., Wen J., Wiley S.E., Worby C.A., Kinch L.N., Xiao J., Grishin N.V., Dixon J.E. (2012). Secreted Kinase Phosphorylates Extracellular Proteins That Regulate Biomineralization. Science.

[10] Manning G., Whyte D.B., Martinez R., Hunter T., Sudarsanam S. (2002). The Protein Kinase Complement of the Human Genome. Science.

[11] Tagliabracci V.S., Wiley S.E., Guo X., Kinch L.N., Durrant E., Wen J., Xiao J., Cui J., Nguyen K.B., Engel J.L. (2015). A Single Kinase Generates the Majority of the Secreted Phosphoproteome. Cell.

[12] Johnson J.L., Yaron T.M., Huntsman E.M., Kerelsky A., Song J., Regev A., Lin T.-Y., Liberatore K., Cizin D.M., Cohen B.M. (2023). An Atlas of Substrate Specificities for the Human Serine/Threonine Kinome. Nature.

[13] Uhlén M., Fagerberg L., Hallström B.M., Lindskog C., Oksvold P., Mardinoglu A., Sivertsson Å., Kampf C., Sjöstedt E., Asplund A. (2015). Proteomics. Tissue-Based Map of the Human Proteome. Science.

[14] Xu R., Tan H., Zhang J., Yuan Z., Xie Q., Zhang L. (2021). Fam20C in Human Diseases: Emerging Biological Functions and Therapeutic Implications. Front. Mol. Biosci..

[15] Simpson M.A., Hsu R., Keir L.S., Hao J., Sivapalan G., Ernst L.M., Zackai E.H., Al-Gazali L.I., Hulskamp G., Kingston H.M. (2007). Mutations in FAM20C Are Associated with Lethal Osteosclerotic Bone Dysplasia (Raine Syndrome), Highlighting a Crucial Molecule in Bone Development. Am. J. Hum. Genet..

[16] Chen X., Zhang J., Liu P., Wei Y., Wang X., Xiao J., Wang C.-C., Wang L. (2021). Proteolytic Processing of Secretory Pathway Kinase Fam20C by Site-1 Protease Promotes Biomineralization. Proc. Natl. Acad. Sci. USA.

[17] Zhang J., Zhu Q., Wang X., Yu J., Chen X., Wang J., Wang X., Xiao J., Wang C.-C., Wang L. (2018). Secretory Kinase Fam20C Tunes Endoplasmic Reticulum Redox State via Phosphorylation of Ero1α. EMBO J..

[18] Pollak A.J., Haghighi K., Kunduri S., Arvanitis D.A., Bidwell P.A., Liu G.-S., Singh V.P., Gonzalez D.J., Sanoudou D., Wiley S.E. (2017). Phosphorylation of Serine96 of Histidine-Rich Calcium-Binding Protein by the Fam20C Kinase Functions to Prevent Cardiac Arrhythmia. Proc. Natl. Acad. Sci. USA.

[19] Pollak A.J., Liu C., Gudlur A., Mayfield J.E., Dalton N.D., Gu Y., Chen J., Heller Brown J., Hogan P.G., Wiley S.E. (2018). A Secretory Pathway Kinase Regulates Sarcoplasmic Reticulum Ca2+ Homeostasis and Protects against Heart Failure. eLife.

[20] Yu J., Li T., Liu Y., Wang X., Zhang J., Wang X., Shi G., Lou J., Wang L., Wang C.-C. (2020). Phosphorylation Switches Protein Disulfide Isomerase Activity to Maintain Proteostasis and Attenuate ER Stress. EMBO J..

[21] Mayfield J.E., Dixon J.E. (2023). Emerging Mechanisms of Regulation for Endoplasmic/Sarcoplasmic Reticulum Ca2+ Stores by Secretory Pathway Kinase FAM20C. Curr. Opin. Chem. Biol..

[22] Noventa F., Salvi M. (2025). Protein Kinase FAM20C-When Subcellular Localization Matters. FEBS Lett..

[23] Tagliabracci V.S., Pinna L.A., Dixon J.E. (2013). Secreted Protein Kinases. Trends Biochem. Sci..

[24] Cui J., Xiao J., Tagliabracci V.S., Wen J., Rahdar M., Dixon J.E. (2015). A Secretory Kinase Complex Regulates Extracellular Protein Phosphorylation. eLife.

[25] Cui J., Zhu Q., Zhang H., Cianfrocco M.A., Leschziner A.E., Dixon J.E., Xiao J. (2017). Structure of Fam20A Reveals a Pseudokinase Featuring a Unique Disulfide Pattern and Inverted ATP-Binding. eLife.

[26] O’Sullivan J., Bitu C.C., Daly S.B., Urquhart J.E., Barron M.J., Bhaskar S.S., Martelli-Júnior H., dos Santos Neto P.E., Mansilla M.A., Murray J.C. (2011). Whole-Exome Sequencing Identifies FAM20A Mutations as a Cause of Amelogenesis Imperfecta and Gingival Hyperplasia Syndrome. Am. J. Hum. Genet..

[27] Hornbeck P.V., Zhang B., Murray B., Kornhauser J.M., Latham V., Skrzypek E. (2015). PhosphoSitePlus, 2014: Mutations, PTMs and Recalibrations. Nucleic Acids Res.

[28] Käll L., Krogh A., Sonnhammer E.L.L. (2007). Advantages of Combined Transmembrane Topology and Signal Peptide Prediction--the Phobius Web Server. Nucleic Acids Res..

[29] O’Shea J.P., Chou M.F., Quader S.A., Ryan J.K., Church G.M., Schwartz D. (2013). pLogo: A Probabilistic Approach to Visualizing Sequence Motifs. Nat. Methods.

[30] Garcia-Moreno A., López-Domínguez R., Villatoro-García J.A., Ramirez-Mena A., Aparicio-Puerta E., Hackenberg M., Pascual-Montano A., Carmona-Saez P. (2022). Functional Enrichment Analysis of Regulatory Elements. Biomedicines.

[31] UniProt Consortium (2025). UniProt: The Universal Protein Knowledgebase in 2025. Nucleic Acids Res.

[32] Benson D.A., Cavanaugh M., Clark K., Karsch-Mizrachi I., Ostell J., Pruitt K.D., Sayers E.W. (2018). GenBank. Nucleic Acids Res..

[33] Huson D.H., Bryant D. (2024). The SplitsTree App: Interactive Analysis and Visualization Using Phylogenetic Trees and Networks. Nat. Methods.

[34] Huson D.H. (1998). SplitsTree: Analyzing and Visualizing Evolutionary Data. Bioinformatics.

[35] Katoh K., Rozewicki J., Yamada K.D. (2019). MAFFT Online Service: Multiple Sequence Alignment, Interactive Sequence Choice and Visualization. Brief. Bioinform..

[36] Kuraku S., Zmasek C.M., Nishimura O., Katoh K. (2013). aLeaves Facilitates On-Demand Exploration of Metazoan Gene Family Trees on MAFFT Sequence Alignment Server with Enhanced Interactivity. Nucleic Acids Res..

[37] Katoh K., Standley D.M. (2013). MAFFT Multiple Sequence Alignment Software Version 7: Improvements in Performance and Usability. Mol. Biol. Evol..

[38] Waterhouse A.M., Procter J.B., Martin D.M.A., Clamp M., Barton G.J. (2009). Jalview Version 2—A Multiple Sequence Alignment Editor and Analysis Workbench. Bioinformatics.

[39] Trifinopoulos J., Nguyen L.-T., von Haeseler A., Minh B.Q. (2016). W-IQ-TREE: A Fast Online Phylogenetic Tool for Maximum Likelihood Analysis. Nucleic Acids Res..

[40] Landrum M.J., Lee J.M., Riley G.R., Jang W., Rubinstein W.S., Church D.M., Maglott D.R. (2014). ClinVar: Public Archive of Relationships among Sequence Variation and Human Phenotype. Nucleic Acids Res..

[41] Rehm H.L., Berg J.S., Brooks L.D., Bustamante C.D., Evans J.P., Landrum M.J., Ledbetter D.H., Maglott D.R., Martin C.L., Nussbaum R.L. (2015). ClinGen—The Clinical Genome Resource. N. Engl. J. Med..

[42] Cozza G., Salvi M. (2018). The Acidophilic Kinases PLK2 and PLK3: Structure, Substrate Targeting and Inhibition. Curr. Protein Pept. Sci..

[43] Crooks G.E., Hon G., Chandonia J.-M., Brenner S.E. (2004). WebLogo: A Sequence Logo Generator. Genome Res..

[44] Cesaro L., Zuliani A.M., Bosello Travain V., Salvi M. (2023). Exploring Protein Kinase CK2 Substrate Recognition and the Dynamic Response of Substrate Phosphorylation to Kinase Modulation. Kinases Phosphatases.

[45] Salvi M., Cesaro L., Pinna L.A. (2010). Variable Contribution of Protein Kinases to the Generation of the Human Phosphoproteome: A Global Weblogo Analysis. Biomol. Concepts.

[46] Cozza G., Moro E., Black M., Marin O., Salvi M., Venerando A., Tagliabracci V.S., Pinna L.A. (2018). The Golgi “casein Kinase” Fam20C Is a Genuine “Phosvitin Kinase” and Phosphorylates Polyserine Stretches Devoid of the Canonical Consensus. FEBS J..

[47] Brunati A.M., Marin O., Bisinella A., Salviati A., Pinna L.A. (2000). Novel Consensus Sequence for the Golgi Apparatus Casein Kinase, Revealed Using Proline-Rich Protein-1 (PRP1)-Derived Peptide Substrates. Biochem. J..

[48] Liu P., Ma S., Zhang H., Liu C., Lu Y., Chen L., Qin C. (2017). Specific Ablation of Mouse Fam20C in Cells Expressing Type I Collagen Leads to Skeletal Defects and Hypophosphatemia. Sci. Rep..

[49] Da Q., Han H., Valladolid C., Fernández M., Khatlani T., Pradhan S., Nolasco J., Matsunami R.K., Engler D.A., Cruz M.A. (2019). In Vitro Phosphorylation of von Willebrand Factor by FAM20c Enhances Its Ability to Support Platelet Adhesion. J. Thromb. Haemost..

[50] Mameli C., Zichichi G., Mahmood N., Elalaoui S.C., Mirza A., Dharmaraj P., Burrone M., Cattaneo E., Sheth J., Gandhi A. (2020). Natural History of Non-Lethal Raine Syndrome during Childhood. Orphanet J. Rare Dis..

[51] Simpson M., Scheuerle A., Hurst J., Patton M., Stewart H., Crosby A. (2009). Mutations in FAM20C Also Identified in Non-Lethal Osteosclerotic Bone Dysplasia. Clin. Genet..

[52] Fradin M., Stoetzel C., Muller J., Koob M., Christmann D., Debry C., Kohler M., Isnard M., Astruc D., Desprez P. (2011). Osteosclerotic Bone Dysplasia in Siblings with a Fam20C Mutation. Clin. Genet..

[53] Zhang R., Ren Y., Ju Y., Zhang Y., Zhang Y., Wang Y. (2025). FAM20C: A Key Protein Kinase in Multiple Diseases. Genes. Dis..

[54] Ben Djoudi Ouadda A., Gauthier M.-S., Susan-Resiga D., Girard E., Essalmani R., Black M., Marcinkiewicz J., Forget D., Hamelin J., Evagelidis A. (2019). Ser-Phosphorylation of PCSK9 (Proprotein Convertase Subtilisin-Kexin 9) by Fam20C (Family With Sequence Similarity 20, Member C) Kinase Enhances Its Ability to Degrade the LDLR (Low-Density Lipoprotein Receptor). Arterioscler. Thromb. Vasc. Biol..

[55] Rallapalli P.M., Orengo C.A., Studer R.A., Perkins S.J. (2014). Positive Selection during the Evolution of the Blood Coagulation Factors in the Context of Their Disease-Causing Mutations. Mol. Biol. Evol..

[56] Zakas P.M., Coyle C.W., Brehm A., Bayer M., Solecka-Witulska B., Radford C.E., Brown C., Nesbitt K., Dwyer C., Kannicht C. (2021). Molecular Coevolution of Coagulation Factor VIII and von Willebrand Factor. Blood Adv..

