# Supplementary material for: Comprehensive Analysis of the Putative Substratome of FAM20C, the Master Serine Kinase of the Secretory Pathway
Source: Biomolecules. 2025 Nov 11;15(11):1582. doi: 10.3390/biom15111582 (PMC12650399; doi:10.3390/biom15111582)
Supplement: Supplementary file 1 [file biomolecules-15-01582-s001.zip › biomolecules-3933793-supplementary.pdf]

Supplementary TABLES

Table S1. Phosphorylation Sites Targeted by FAM20C (Full-Length Proteins or Peptides) This table lists sites phosphorylated by FAM20C, either in full-length proteins or peptides. Phosphorylated peptide sequences are shown in a ±7 amino acid window around the modified residue (gaps may appear if the full window is not present in the peptide). Abbreviations- Acc. numb.: Accession Number; Cell Comp.: Cell Component; S: specie; Ref: Reference; C: cow; H: human, R: rat. ER-l: ER lumen; ER-m: ER membrane with the phosphosite located in the lumen; Golgi-m: Golgi membrane, with the phosphosite located in the lumen; ER/Golgi: ER or Golgi membrane, with the phosphosite located in the lumen; PM: plasma membrane protein, with the phosphosite located in the extracellular domain.

Table S2. Compilation of S-X-E Motif Phosphosites in Human Secretory Pathway Proteins

Phosphosites were extracted from the PhosphoSitePlus database and include only those containing the FAM20C consensus sequence (S-X-E) and mapped to proteins localized in the endoplasmic reticulum (ER) lumen, Golgi lumen, or extracellular space. The correct localization within the secretory pathway was manually verified. For membrane proteins, the localization of phosphosites in luminal domains or in extracellular domains was confirmed by checking the topology in the UniProt database or by using the Phobius prediction tool [1]. We also excluded phosphosites from proteins secreted extracellularly via non-secretory pathways.Phosphosites and/or proteins highlighted in red have already been identified as FAM20C substrates (see Tables S1 and S5 for phosphosites, Table S3 and S4 for proteins). Abbreviations- Acc. numb.: Accession Number; Cell Comp.: Cell Component; ER-l: ER lumen; ER-m: ER membrane with the phosphosite located in the lumen; Golgi-m: Golgi membrane, with the phosphosite located in the lumen; ER/Golgi: ER or Golgi membrane, with the phosphosite located in the lumen; Perinuc: perinuclear; PM: plasma membrane protein, with the phosphosite located in the extracellular domain.

Table S3. Direct protein Substrates of FAM20C Identified in Vitro and/or in Cells This table lists proteins that are direct substrates of FAM20C, as demonstrated through in vitro assays and/or cellular experiments. Proteins for which the specific phosphorylation site(s) have not yet been identified are highlighted in red. Abbreviations- Acc. numb.: Accession Number; Cell Comp.: Cell Component; Ref: Reference. ER-l: ER lumen.

Table S4. Phosphorylation Sites decreased in FAM20C Knockout (KO) or Knockdown (KD) Human Cells

This table presents phosphosites with reduced phosphorylation levels in human cells following FAM20C knockout or knockdown. Dara are from [2]. Only sites containing the FAM20C consensus sequence (SXE) are included, suggesting they are likely direct targets of FAM20C activity. Abbreviations- Acc. numb.: Accession Number; Cell Comp.: Cell Component; ER-l: ER lumen; ER-m: ER membrane with the phosphosite located in the lumen; Golgi-m: Golgi membrane, with the phosphosite located in the lumen; ER/Golgi: ER or Golgi membrane, with the phosphosite located in the lumen; PM: plasma membrane protein, with the phosphosite located in the extracellular domain.

Table S1.

Acc. numb.	Gene name	Protein name	S	Target	pSite	Sequence	Cell Comp.	Ref
P02765	AHSG	Alpha-2-HS-glycoprotein (FETUA)	H	Protein	S330	VVSLGSPsGEVSHPR	Secreted	[3]
P05067	APP	Amyloid beta precursor protein	H	Peptide	S441	______VESLEQEAAN	PM	[6]
O95972	BMP15	Bone morphogenetic protein 15	H	Protein	S273	TRQADGISAEVTASS	Secreted	[4]
O14958	CASQ2	Calsequestrin 2	H	Protein	S385	DDDDDDNsDEEDNDD	ER-l	[5]
P05060	CHGB	Secretogranin 1	H	Peptide	S405	__GYGEESEEER___	Secreted	[6]
P02662	CSN1S1	Alpha-S1-casein	C	Protein	S61	ELSKDIGSESTEDQA	Secreted	[7]
P02662	CSN1S1	Alpha-S1-casein	C	Protein	S63	SKDIGSESTEDQAME	Secreted	[7]
P02662	CSN1S1	Alpha-S1-casein	C	Protein	S79	IKQMEAESISSSEEI	Secreted	[7]
P02662	CSN1S1	Alpha-S1-casein	C	Protein	S81	QMEAESISSSEEIVP	Secreted	[7]
P02662	CSN1S1	Alpha-S1-casein	C	Protein	S82	MEAESISSSEEIVPN	Secreted	[7]
P02662	CSN1S1	Alpha-S1-casein	C	Protein	S83	EAESISSSEEIVPNS	Secreted	[7]
P02662	CSN1S1	Alpha-S1-casein	C	Protein	S90	SEEIVPNSVEQKHIQ	Secreted	[7]
P02662	CSN1S1	Alpha-S1-casein	C	Protein	S130	QLEIVPNSAEERLHS	Secreted	[7]
P02666	CSN2	β-casein	C	Peptide	S33	EIVESLSsSEESITR	Secreted	[8]
P02666	CSN2	β-casein	C	Peptide	S34	IVESLSSsEESITRI	Secreted	[8]
P02666	CSN2	β-casein	C	Peptide	S50	KKIEKFQsEEQQQTE	Secreted	[9]
P01034	CST3	Cystatin-C	H	Peptide	S43	_____MDASVEEEGVR	Secreted	[6]
Q96HE7	ERO1A	ERO1-like protein alpha	H	Protein	S145	GAVDEsLsEETQkAV	ER-l	[10]
O94769	ECM2	Extracellular matrix protein 2	H	Peptide	S213	____EALQsEEDEEVK	Secreted	[6]
Q8IXL6	FAM20C	Extracellular serine/threonine protein kinase FAM20C	H	Protein	S106	SsNLSSHsLEKLPPA	Golgi-l	[11]
P02671	FGA	Fibrinogen alpha chain	H	Peptide	S22	GTAWTADsGEGDFLA	Secreted	[8]
Q9GZV9	FGF23	Fibroblast growth factor 23	H	Protein	S180	IPRRHtRsAEDDSER	Secreted	[12]
Q9GZV9	FGF23	Fibroblast growth factor 23	H	Protein	S207	TPAPASCsQELPsAE	Secreted	[12]
Q9GZV9	FGF23	Fibroblast growth factor 23	H	Protein	S212	SCsQELPsAEDNSPM	Secreted	[12]
Q9EPC2	Fgf23	Fibroblast growth factor 23	M	Protein	S212	SCSRELPsAEEGGPA	Secreted	[12]
O60383	GDF9	Growth/differentiation factor 9	H	Protein	S325	RRGQETVSSELKKPL	Secreted	[4]
Q8NBJ4	GOLM1	Golgi phosphoprotein 2	H	Peptide	T255	________TNEIQVVN	Golgi-m	[6]
P15515	HTN1	Histatin 1	H	Peptide	S21	ISMISADSHEKRHHG	Secreted	[13]
P23327	HRC	Sarcoplasmic reticulum histidine-rich calcium-binding protein	H	Protein	S96	EKEDEDVsKEYGHLL	ER-l	[14]
P08833	IGFBP1	Insulin-like growth factor-binding protein 1	H	Protein	S45	LALCPPVsASCSEVT	Secreted	[2]
P08833	IGFBP1	Insulin-like growth factor-binding protein 1	H	Protein	S194	LAKAQETsGEEIsKF	Secreted	[2]
P08833	IGFBP1	Insulin-like growth factor-binding protein 1	H	Protein	S199	ETsGEEIsKFYLPNC	Secreted	[2]
Q9NQ76	MEPE	Matrix extracellular phosphoglycoprotein	H	Protein	S78	DLSLSEAsENKGSSK	Secreted	[15]
Q9NQ76	MEPE	Matrix extracellular phosphoglycoprotein	H	Protein	S239	IPSDFEGsGYTDLQE	Secreted	[15]
Q9NQ76	MEPE	Matrix extracellular phosphoglycoprotein	H	Protein	S379	FHYPPAPsKEKRKEG	Secreted	[15]

Q9NQ76	MEPE	Matrix extracellular phosphoglycoprotein	H	Protein	S458	MDSFNGPsHENIITH	Secreted	[15]
P10451	SPP1	Osteopontin	H	Protein	S162	TYDGRGDsVVYGLRS	Secreted	[16]
Q16549	PCSK7	Proprotein convertase subtilisin/kexin type 7	H	Protein	S505	AIPQSPRsLEVLWNV	Golgi-m	[17]
Q8NBP7	PCSK9	Proprotein convertase subtilisin/kexin type 9	H	Protein	S47	ELVLALRsEEDGLAE	Secreted	[18]
Q8NBP7	PCSK9	Proprotein convertase subtilisin/kexin type 9	H	Protein	S666	CDVSTTGsTSEEAVT	Secreted	[18]
Q8NBP7	PCSK9	Proprotein convertase subtilisin/kexin type 9	H	Protein	S668	VSTTGSTsEEAVTAV	Secreted	[18]
Q8NBP7	PCSK9	Proprotein convertase subtilisin/kexin type 9	H	Protein	S688	SRHLAQAsQELQ	Secreted	[18]
P07237	P4HB	Protein disulfide-isomerase	H	Protein	S357	KIKPHLMsQELPEDW	ER-l	[19]
P02810	PRH1	Salivary acidic proline-rich phosphoprotein 1/2	H	Peptide	S24	QDLDEDVSQEDVPLV	Secreted	[13]
P02810	PRH1	Salivary acidic proline-rich phosphoprotein 1/2	H	Peptide	S38	VISDGGDSEQFIDEE	Secreted	[13, 20]
Q92932	PTPRN2	Receptor-type tyrosine-protein phosphatase N2	H	Peptide	S360	__AALGESGEQADGP	Secretory vesicle-m	[6]
Q8WXD2	SCG3	Secretogranin 3	H	Peptide	S37	______ELSAERPLNE	Secreted	[6]
P05408	Scg5	Neuroendocrine protein 7B2	R	Protein	T98	PNIVAELtGDNIPKD	Secreted	[21]
P05408	Scg5	Neuroendocrine protein 7B2	R	Protein	T135	CLENAPDtAEFsREF	Secreted	[21]
Q9BRK5	SDF4	45 kDa calcium-binding protein	H	Protein	T193	ELkVDEEtQEVLENL	Golgi/ER-l	[22]
Q9BRK5	SDF4	45 kDa calcium-binding protein	H	Protein	S349	YSEFFtGskLVDYAR	Golgi/ER-l	[22]
Q9UK55	SERPINA10	Protein Z-dependent protease inhibitor	H	Peptide	S56	EEDEQEASEEK_____	Secreted	[6]
Q14515	SPARCL1	SPARC like protein 1	H	Peptide	S198	_____PNISNGEEEEE	Secreted	[6]
P02808	STATH	Statherin	H	Peptide	S21	VSMIGADSSEEKFLR	Secreted	[13]
P02808	STATH	Statherin	H	Peptide	S22	SMIGADSSEEKFLRR	Secreted	[13]
Q13586	STIM1	Stromal interaction molecule 1	H	Protein	S88	GDVDVEESDEFLRED	ER-m	[5]
P04004	VTN	Vitronectin	H	Peptide	S312	_______DSWEDIFEL	Secreted	[6]
P04275	VWF	von Willebrand factor	H	Protein	S1517	GEADFNRsKEFMEEV	Secreted	[23]
P04275	VWF	von Willebrand factor	H	Protein	S1613	MVTGNPAsDEIKRLP	Secreted	[23]

Table S2.

Acc. numb.	Gene name	Protein name	pSite	Sequence	Cell comp.
P58397	ADAMTS12	A disintegrin and metalloproteinase with thrombospondin motifs 12	S436	PLtWsKCsEEYITRF	Secreted
Q9UKP4	ADAMTS7	A disintegrin and metalloproteinase with thrombospondin motifs 7	S59	DAGGSFLsyELWPRA	Secreted
Q8N6G6	ADAMTSL 1	ADAMTS-like protein 1	S976	ARPLsPRsEEEVLAG	Secreted
P82987	ADAMTSL 3	ADAMTS-like protein 3	S631	ACDESPAsRELDIPL	Secreted
Q6UY14	ADAMTSL 4	ADAMTS-like protein 4	S696	FLCISREsGEELDER	Secreted
P02765	AHSG	Alpha-2-HS-glycoprotein	S138	kCDssPDsAEDVrkV	Secreted
P02765	AHSG	Alpha-2-HS-glycoprotein	S330	VVsLGsPsGEVsHPR	Secreted
P02765	AHSG	Alpha-2-HS-glycoprotein	S74	KVWPQQPsGELFEIE	Secreted
P02768	ALB	Albumin	S82	kTCVADEsAENCDKs	Secreted
P10696	ALPPL2	Alkaline phosphatase, germ cell type	S365	ERAGQLtsEEDtLSL	PM
P10696	ALPPL2	Alkaline phosphatase, germ cell type	S435	VtEsEsGsPEyRQQS	PM
Q15389	ANGPT1	Angiopoietin-1	S25	GCSNQRRsPENSGRR	Secreted
Q9UKU9	ANGPTL2	Angiopoietin-related protein 2	S73	TGAICVNsKEPEVLL	Secreted
Q9HCE9	ANO8	Anoctamin-8	S1032	LSFKFLKsPETRRDS	PM
Q06481	APLP2	Amyloid beta precursor like protein 2	S213	TKIIGsVskEEEEED	PM
P02647	APOA1	Apolipoprotein A-I	S191	RtHLAPysDELRQRL	Secreted
P02652	APOA2	Apolipoprotein A-II	S68	AKSYFEksKEQLTPL	Secreted
P02652	APOA2	Apolipoprotein A-II	S54	DLMEKVKsPELQAEA	Secreted
P06727	APOA4	Apolipoprotein A-IV	S259	LKARIsAsAEELRQR	Secreted
Q6Q788	APOA5	Apolipoprotein A-V	S59	EPAtLKDsLEQDLNN	Secreted
P04114	APOB	Apolipoprotein B-100	S4048	tELRVREsDEEtQIK	Secreted
P02649	APOE	Apolipoprotein E	S147	VQAMLGQsTEELRVR	Secreted
O14791	APOL1	Apolipoprotein L1	S314	tEPIsAEsGEQVERV	Secreted
O14791	APOL1	Apolipoprotein L1	S311	PRVtEPIsAEsGEQV	Secreted
Q9BQE5	APOL2	Apolipoprotein L2	S250	PHVIGRIsAEGGEQV	Secreted
P05067	APP	Amyloid-beta precursor protein	S441	HFQEKVEsLEQEAAN	PM
O95972	BMP15	Bone morphogenetic protein 15	S273	TRQADGIsAEVTASS	Secreted
Q8N4F0	BPIFB2	BPI fold-containing family B member 2	S60	VPHFLDWsGEALQPT	Secreted
Q8N4F0	BPIL1	BPI fold-containing family B member 2	S60	VPHFLDWsGEALQPT	Secreted
Q9NX62	BPNT2	Golgi-resident adenosine 3',5'-bisphosphate 3'-phosphatase	S283	LLDVPDksQEKADLY	Golgi-l
Q9H972	C14orf93	Uncharacterized protein C14orf93	S428	DVTEELMsDEEDSLN	Secreted
Q9BUN1	C1orf56	Protein MENT	S106	AINEEDGssEEGVVI	Secreted
Q9BUN1	C1orf56	Protein MENT	S107	INEEDGssEEGVVIN	Secreted
Q9BUN1	C1orf56	Protein MENT	S143	STRFIANsQEPEIRL	Secreted
P01024	C3	Complement C3	S297	RIPIEDGsGEVVLsR	Secreted
P01024	C3	Complement C3	S1321	EsASLLRsEETKENE	Secreted
P01024	C3	Complement C3	S1573	EQTIKSGsDEVQVGQ	Secreted
P04003	C4BPA	C4b-binding protein alpha chain	S187	DIRNGRHsGEENFYA	Secreted
P01031	C5	Complement C5	S662	TNANADDsQENDEPC	Secreted
P10643	C7	Complement component C7	S331	LKQNDFNsVEEKKCK	Secreted
P07357	C8A	Complement component C8 alpha chain	S125	DQDCLDGsDEDDCED	Secreted
P07358	C8B	Complement component C8 beta chain	S150	DNDCGDQsDEANCRR	Secreted

P02748	C9	Complement component C9	S66	RQMFRSRsIEVFGQF	Secreted
P27797	CALR	Calreticulin	S80	rFyALsAsFEPFsNK	ER-l
P27797	CALR	Calreticulin	S195	NSQVESGsLEDDWDF	ER-l
O43852	CALU	Calumenin	S69	DQLtPEEskERLGkI	Er/Golgi -l
O43852	CALU	Calumenin	S125	LNEDGLVsWEEyKNA	Er/Golgi -l
Q8WVQ1	CANT1	Soluble calcium-activated nucleotidase 1	S303	FFLPRRAsQERYSEK	Er/Golgi -m
Q16568	CARTPT	Cocaine- and amphetamine-regulated transcript protein	S48	YsAVDDAsHEKELIE	Secreted
O95389	CCN6	Cellular communication network factor 6	S180	KSDQSNCsLEPLLQQ	Secreted
P28906	CD34	Hematopoietic progenitor cell antigen CD34	S34	ySsGPGTsPEAQGKA	PM
P19022	CDH2	Cadherin-2	S135	TEESVKEsAEVEEIV	PM
P08603	CFH	Complement factor H	S1196	LYSRTGEsVEFVCKR	Secreted
P36980	CFHR2	Complement factor H-related protein 2	S145	PKCRSTIsAEKCGPP	Secreted
P36980	CFHR2	Complement factor H-related protein 2	S210	CLDPCVIsQEIMEKY	Secreted
P05156	CFI	Complement factor I	S90	PTYCQQKsLECLHPG	Secreted
P10645	CHGA	Chromogranin-A	S402	RPSsREDsLEAGLPL	Secreted
P10645	CHGA	Chromogranin-A	S333	EQEEERLsKEWEDSK	Secreted
P10645	CHGA	Chromogranin-A	S322	GLFRGGKsGELEQEE	Secreted
P10645	CHGA	Chromogranin-A	S398	RRGWRPSsREDsLEA	Secreted
P10645	CHGA	Chromogranin-A	S218	VDREKGLsAEPGWQA	Secreted
P10645	CHGA	Chromogranin-A	S142	DsKEAEKsGEAtDGA	Secreted
P10645	CHGA	Chromogranin-A	S136	VMEKREDsKEAEKsG	Secreted
P10645	CHGA	Chromogranin-A	S53	KPSPMPVsQECFETL	Secreted
P05060	CHGB	Secretogranin-1	S405	AHGyGEEsEEERGLE	Secreted
P05060	CHGB	Secretogranin-1	S130	WAEGGGHsRERADEP	Secreted
P05060	CHGB	Secretogranin-1	S149	YPSDSQVsEEVKTRH	Secreted
P05060	CHGB	Secretogranin-1	S335	HSTHyRAsEEEPEyG	Secreted
P05060	CHGB	Secretogranin-1	S301	SQGGSLPsEEKGHPQ	Secreted
P05060	CHGB	Secretogranin-1	S626	EFPDFyDsEEPVSTH	Secreted
P05060	CHGB	Secretogranin-1	S377	YRAPRPQsEEsWDEE	Secreted
P05060	CHGB	Secretogranin-1	S311	KGHPQEEsEESNVsM	Secreted
P05060	CHGB	Secretogranin-1	S259	ESKGQPRsQEEsEEG	Secreted
P05060	CHGB	Secretogranin-1	S263	QPRsQEEsEEGEEDA	Secreted
P05060	CHGB	Secretogranin-1	S391	EDKRNYPsLELDKMA	Secreted
P05060	CHGB	Secretogranin-1	S367	ERYRGRGsEEYRAPR	Secreted
P05060	CHGB	Secretogranin-1	S617	LLHyRKKsAEFPDFy	Secreted
P05060	CHGB	Secretogranin-1	S182	KGERGEDssEEKHLE	Secreted
P05060	CHGB	Secretogranin-1	S183	GERGEDssEEKHLEE	Secreted
Q9BU40	CHRDL1	Chordin-like protein 1	S185	CRGDGELsWEHSDGD	Secreted
Q07065	CKAP4	Cytoskeleton-associated protein 4	S460	GRLEGLGssEADQDG	ER-m/perinuc
Q07065	CKAP4	Cytoskeleton-associated protein 4	S232	ARERDFtsLENtVEE	ER-m/perinuc
O75596	CLEC3A	C-type lectin domain family 3 member A	S108	ILVIPRNsDEINALQ	Secreted
Q9BT09	CNPY3	Protein canopy homolog 3	S257	GGLEGDPsPEEDEGI	ER-l
Q8N129	CNPY4	Protein canopy homolog 4	S58	ELSRTGRsREVLELG	Secreted
P39059	COL15A1	Collagen alpha-1(XV) chain	S976	VDTAHPGsPELITFH	Secreted
Q07092	COL16A1	Collagen alpha-1(XVI) chain	S260	KARRDtQsNELIEIN	Secreted
Q9UMD9	COL17A1	Collagen alpha-1(XVII) chain	S640	GEAGPPGsGEKGERG	Secreted
Q9UMD9	COL17A1	Collagen alpha-1(XVII) chain	S544	KIGLHsDsQEELWMF	Secreted
P39060	COL18A1	Collagen alpha-1(XVIII) chain	S705	PPLAGGsstEDsRsE	Secreted
P39060	COL18A1	Collagen alpha-1(XVIII) chain	S711	sstEDsRsEEVEEQT	Secreted
P02452	COL1A1	Collagen alpha-1(I) chain	S1247	QQIENIRsPEGSRKN	Secreted

P02452	COL1A1	Collagen alpha-1(I) chain	S1271	MCHSDWksGEYWIDP	Secreted
P02452	COL1A1	Collagen alpha-1(I) chain	S1393	KALLLQGsNEIEIRA	Secreted
P12110	COL6A2	Collagen alpha-2(VI) chain	S223	YATMLPDsTEIDQDT	Secreted
P12111	COL6A3	Collagen alpha-3(VI) chain	S1783	VGVRNIDsEEVGKIA	Secreted
P12111	COL6A3	Collagen alpha-3(VI) chain	S162	KDGLALPsAELKSAD	Secreted
A8TX70	COL6A5	Collagen alpha-5(VI) chain	S2255	NYEKDQKsAEIASLT	Secreted
Q02388	COL7A1	Collagen alpha-1(VII) chain	S828	ILPGNTDsAEIRGLE	Secreted
O75976	CPD	Carboxypeptidase D	S1361	GsKksLLsHEFQDEt	PM
Q6UXH1	CRELD2	Protein disulfide isomerase CRELD2	S70	kTLSKyEssEIRLLE	ER-l
P47710	CSN1S1	Alpha-S1-casein	S90	MESsIssssEEMSLS	Secreted
P47710	CSN1S1	Alpha-S1-casein	S91	ESsIssssEEMSLSK	Secreted
P47710	CSN1S1	Alpha-S1-casein	S41	SEPIPLEsREEYMNG	Secreted
P47710	CSN1S1	Alpha-S1-casein	S33	RLQNPsEsSEPIPLE	Secreted
P05814	CSN2	Beta-casein	S24	EtIEsLsssEESITE	Secreted
P05814	CSN2	Beta-casein	S25	tIEsLsssEESITEY	Secreted
P13611	CSPG2	Versican core protein	S1351	GHPIDsEsKEDEPCS	Secreted
P13611	CSPG2	Versican core protein	S2116	EIESETtsEEQIQEE	Secreted
P13611	CSPG2	Versican core protein	S2941	NKTDGQVsGEAIKMF	Secreted
P13611	CSPG2	Versican core protein	S3209	AHLtsILsHEEQMFV	Secreted
P01034	CST3	Cystatin-C	S43	VGGPMDAsVEEEGVR	Secreted
P01036	CST4	Cystatin-S	S23	AGALAsSsKEENRII	Secreted
P01036	CST4	Cystatin-S	S119	LQKKQLCsFEIYEVP	Secreted
Q9H4G1	CST9L	Cystatin-9-like	S88	VESKTVFsMELLLGR	Secreted
P07339	CTSD	Cathepsin D	S42	TMsEVGGsVEDLIAk	Secreted/endosome-l
P07108	DBI	Acyl-CoA-binding protein	S21	RHLkTkPsDEEMLFI	ER/Golgi-l
P80370	DLK1	Protein delta homolog 1	S355	NLLLQyNsGEDLAVN	PM
Q9UBS3	DNAJB9	DnaJ homolog subfamily B member 9	S106	GQRGsGSsFEQSFNF	ER-l
Q13217	DNAJC3	DnaJ homolog subfamily C member 3	S274	KLNkLIEsAEELIRD	ER-l
O94769	ECM2	Extracellular matrix protein 2	S213	VRKEALQsEEDEEVK	Secreted
O94769	ECM2	Extracellular matrix protein 2	S75	PIVNFDysMEEKFES	Secreted
Q9BZQ6	EDEM3	ER degradation-enhancing alpha-mannosidase-like protein 3	S835	DLVDQESsEENSLNS	ER-l
P00533	EGFR	Epidermal growth factor receptor	S511	QVCHALCsPEGCWGP	PM
Q9Y6C2	EMILIN1	EMILIN-1	S703	ATEHATEsEERFRGL	Secreted
P17813	ENG	Endoglin	S634	VAVAAPAssESSStN	PM
Q96HE7	ERO1A	ERO1-like protein alpha	S106	CAVKPCQsDEVPDGI	ER-l
Q96HE7	ERO1A	ERO1-like protein alpha	S145	GAVDEsLsEETQkAV	ER-l
Q96HE7	ERO1A	ERO1-like protein alpha	S121	KSASYKYsEEANNLI	ER-l
Q9H8M9	EVA1A	Protein eva-1 homolog A	S114	LNkNVFtsAEELERA	PM
P03951	F11	Coagulation factor XI	S17	FILFTSVsGECVtQL	Secreted
P05160	F13B	Coagulation factor XIII B chain	S373	SGYLLHGsNEITCNR	Secreted
P12259	F5	Coagulation factor V	S955	HLASEKGsYEIIQDT	Secreted
P12259	F5	Coagulation factor V	S1150	DPSHRsSsPELSEML	Secreted
P12259	F5	Coagulation factor V	S859	LGAGEFKsQEHAkHk	Secreted
P12259	F5	Coagulation factor V	S692	IPDDDEDsyEIFEPP	Secreted
P00740	F9	Coagulation factor IX	S204	PDVDYVNsTEAETIL	Secreted
P00740	F9	Coagulation factor IX	S114	SCKDDINsYECWCPF	Secreted
P56851	FAM12B	Epididymal secretory protein E3-beta	S42	KQHyLsPsREFREYK	Secreted
Q8IXL6	FAM20C	Extracellular serine/threonine protein kinase FAM20C	S106	SsNLSSHsLEKLPPA	Golgi-l
P23142	FBLN1	Fibulin-1	S147	FQACCVKsQETGDLD	Secreted
P23142	FBLN1	Fibulin-1	S100	TPHGDNAsLEATFVK	Secreted
P98095	FBLN2	Fibulin-2	S277	ARRVTEDsEEEEEEE	Secreted
P35555	FBN1	Fibrillin-1	S2702	GNPEPPVsGEMDDNs	Secreted

Q8NFU4	FDCSP	Follicular dendritic cell secreted peptide	S34	EKRSISDsDELASGF	Secreted
Q9UGM5	FETUB	Fetuin-B	S315	PDLDDKNsQEKGPQE	Secreted
P02671	FGA	Fibrinogen alpha chain	S364	TGTWNPGssERGSAG	Secreted
P02671	FGA	Fibrinogen alpha chain	S22	GTAWTADsGEGDFLA	Secreted
P02671	FGA	Fibrinogen alpha chain	S56	DsDWPFCsDEDWNYk	Secreted
P02675	FGB	Fibrinogen beta chain	S173	ENVVNEyssELEKHQ	Secreted
P02675	FGB	Fibrinogen beta chain	S425	SDPRKQCsKEDGGGW	Secreted
Q9GZV9	FGF23	Fibroblast growth factor 23	S180	IPRRHtRsAEDDSER	Secreted
Q9GZV9	FGF23	Fibroblast growth factor 23	S212	SCsQELPsAEDNSPM	Secreted
Q9GZV9	FGF23	Fibroblast growth factor 23	S207	TPAPASCsQELPsAE	Secreted
P22607	FGFR3	Fibroblast growth factor receptor 3	S424	FPLKRQVsLESNASM	PM
P22607	FGFR3	Fibroblast growth factor receptor 3	S445	VRIARLssGEGPtLA	PM
Q96AY3	FKBP10	Peptidyl-prolyl cis-trans isomerase FKBP10	S571	VDELkLKsDEDEERV	ER-l
P02751	FN1	Fibronectin	S2432	RRPGGEPsPEGttGQ	Secreted
Q4ZHG4	FNDC1	Fibronectin type III domain-containing protein 1	S1083	QGsYDDDsTEVEAQD	Secreted
Q4ZHG4	FNDC1	Fibronectin type III domain-containing protein 1	S1176	SKSQQsVsAEDDEEE	Secreted
Q4ZHG4	FNDC1	Fibronectin type III domain-containing protein 1	S255	ALtKRKIsEEDELDV	Secreted
Q4ZHG4	FNDC1	Fibronectin type III domain-containing protein 1	S548	TGEEELGsREDSPMS	Secreted
Q12841	FSTL1	Follistatin-related protein 1	S165	NGDSRLDssEFLKFV	Secreted
O95633	FSTL3	Follistatin-related protein 3	S255	EEPPGGEsAEEEENF	Secreted
Q6MZW2	FSTL4	Follistatin-related protein 4	S485	PtEKIFMsYEEICPQ	Secreted
P22466	GAL	Galanin peptides	S116	LDLPAAAssEDIERs	Secreted
Q14697	GANAB	Neutral alpha-glucosidase AB	S345	MMDyLQGsGEtPQtD	ER/Golgi-l
P02774	GC	Vitamin D-binding protein	S95	TSALSAKsCESNSPF	Secreted
P01275	GCG	Pro-glucagon	S152	RHADGsFsDEMNTIL	Secreted
O60383	GDF9	Growth/differentiation factor 9	S325	RRGQETVsSELKKPL	Secreted
O00461	GOLIM4	Golgi integral membrane protein 4	S364	LEEEHDPsPEEQDRE	Golgi-m
Q8NBJ4	GOLM1	Golgi membrane protein 1	S309	VQAALsVsQENPEME	Golgi-m
Q8TED1	GPX8	Glutathione peroxidase 8	S61	DAKGRTVsLEkYKGK	ER-m
O60565	GREM1	Gremlin-1	S77	GEEVLEssQEALHVT	Secreted
Q9H772	GREM2	Gremlin-2	S56	IKEVLASsQEALVVT	Secreted
P14625	GRP94	Endoplasmin	S227	QHIWEsDsNEFSVIA	ER-l
P14625	GRP94	Endoplasmin	S347	kPIWQRPskEVEEDE	ER-l
P14625	GRP94	Endoplasmin	S447	DDLPLNVsRETLQQH	ER-l
P06396	GSN	Gelsolin	S261	GRARVHVsEEGtEPE	Secreted
Q96S86	HAPLN3	Hyaluronan and proteoglycan link protein 3	S130	QDKEHDVsLEIQDLR	Secreted
P04439	HLA-A	HLA class I histocompatibility antigen, A alpha chain	S275	WAAVVVPsGEEQRYT	PM
P01889	HLA-B	HLA class I histocompatibility antigen, B alpha chain	S275	WAAVVVPsGEEQRYT	PM
P13747	HLA-E	HLA class I histocompatibility antigen, alpha chain E	S272	WAAVVVPsGEEQRYT	PM
P17693	HLA-G	HLA class I histocompatibility antigen, alpha chain G	S275	WAAVVVPsGEEQRYT	PM
Q8NDA2	HMCN2	Hemicentin-2	S2756	SAAFEILsREEEARG	Secreted
P23327	HRC	Sarcoplasmic reticulum histidine-rich calcium-binding protein	S119	KVGDEGVsGEEVFAE	ER-l
P23327	HRC	Sarcoplasmic reticulum histidine-rich calcium-binding protein	S431	REEDEEVsAELGHQA	ER-l
P23327	HRC	Sarcoplasmic reticulum histidine-rich calcium-binding protein	S170	EDEDEVVssEHHHHI	ER-l

P23327	HRC	Sarcoplasmic reticulum histidine-rich calcium-binding protein	S567	APLsPDHsEEEEEEE	ER-l
P23327	HRC	Sarcoplasmic reticulum histidine-rich calcium-binding protein	S145	GsEDtEDsAEHRHHL	ER-l
P23327	HRC	Sarcoplasmic reticulum histidine-rich calcium-binding protein	S333	KEEVEAVsGEHHHHV	ER-l
P23327	HRC	Sarcoplasmic reticulum histidine-rich calcium-binding protein	S206	EEEEEEAstEyGHQA	ER-l
P23327	HRC	Sarcoplasmic reticulum histidine-rich calcium-binding protein	S96	EKEDEDVsKEyGHLL	ER-l
P23327	HRC	Sarcoplasmic reticulum histidine-rich calcium-binding protein	S494	EKEEDPGsHEEDDEs	ER-l
P23327	HRC	Sarcoplasmic reticulum histidine-rich calcium-binding protein	S311	DNDDDDVstEYGHQA	ER-l
P23327	HRC	Sarcoplasmic reticulum histidine-rich calcium-binding protein	S358	EEEDEDVstERWHQG	ER-l
P23327	HRC	Sarcoplasmic reticulum histidine-rich calcium-binding protein	S221	HRHRGHGsEEDEDVs	ER-l
P23327	HRC	Sarcoplasmic reticulum histidine-rich calcium-binding protein	S299	DPsHRHRsHEEDDND	ER-l
P23327	HRC	Sarcoplasmic reticulum histidine-rich calcium-binding protein	S601	REEAGGAsSEEESGE	ER-l
P23327	HRC	Sarcoplasmic reticulum histidine-rich calcium-binding protein	S401	HQPRGHKsDEEDFQD	ER-l
P23327	HRC	Sarcoplasmic reticulum histidine-rich calcium-binding protein	S263	DDDDDDVsIEYRHQA	ER-l
P23327	HRC	Sarcoplasmic reticulum histidine-rich calcium-binding protein	S501	sHEEDDEsSEQGEKG	ER-l
P23327	HRC	Sarcoplasmic reticulum histidine-rich calcium-binding protein	S54	AGLSEEAsAELRHHL	ER-l
Q53GQ0	HSD17B12	Very-long-chain 3-oxoacyl-CoA reductase	S251	kPTLDKPsPETFVkS	ER-m
P98160	HSPG2	Basement membrane-specific heparan sulfate proteoglycan core protein	S2986	HLVSPADsGEYVCRA	Secreted
P98160	HSPG2	Basement membrane-specific heparan sulfate proteoglycan core protein	S2402	YQAsPADsGEYVCRV	Secreted
P98160	HSPG2	Basement membrane-specific heparan sulfate proteoglycan core protein	S105	PQLEDAGsREFREVS	Secreted
P98160	HSPG2	Basement membrane-specific heparan sulfate proteoglycan core protein	S2112	PQVSPADsGEYVCRV	Secreted
P98160	HSPG2	Basement membrane-specific heparan sulfate proteoglycan core protein	S2691	HQMSVADsGEYVCRA	Secreted
P15515	HTN1	Histatin-1	S21	ISMISADsHEKRHHG	Secreted
P83110	HTRA3	Serine protease HTRA3	S214	VQLQNGDsYEATIKD	Secreted
Q9Y4L1	HYOU1	Hypoxia up-regulated protein 1	S567	FETLVEDsAEEESTL	ER-l
Q9Y4L1	HYOU1	Hypoxia up-regulated protein 1	S763	QPEyQEVsTEEQREE	ER-l
Q9Y4L1	HYOU1	Hypoxia up-regulated protein 1	S742	EREkAANsLEAFIFE	ER-l
P21815	IBSP	Bone sialoprotein 2	S31	RRVKIEDsEENGVFK	Secreted
P01579	IFNG	Interferon gamma	S92	DDQSIQKsVETIKED	Secreted
P08833	IGFBP1	Insulin-like growth factor-binding protein 1	S194	LAKAQETsGEEIsKF	Secreted
P08833	IGFBP1	Insulin-like growth factor-binding protein 1	S144	NFHLMAPsEEDHSIL	Secreted
P08833	IGFBP1	Insulin-like growth factor-binding protein 1	S126	GsPEsPEstEITEEE	Secreted

P08833	IGFBP1	Insulin-like growth factor-binding protein 1	S123	AEAGsPEsPEstEIT	Secreted
P08833	IGFBP1	Insulin-like growth factor-binding protein 1	S120	PHAAEAGsPEsPEst	Secreted
P18065	IGFBP2	Insulin-like growth factor-binding protein 2	S142	RDAEYGAsPEQVADN	Secreted
P17936	IGFBP3	Insulin-like growth factor-binding protein 3	S201	stDtQNFsSESKRET	Secreted
P17936	IGFBP3	Insulin-like growth factor-binding protein 3	S148	EEDRsAGsVEsPsVs	Secreted
P17936	IGFBP3	Insulin-like growth factor-binding protein 3	S140	APGNAsEsEEDRsAG	Secreted
P24593	IGFBP5	Insulin-like growth factor-binding protein 5	S116	QVKIERDsREHEEPt	Secreted
Q16270	IGFBP7	Insulin-like growth factor-binding protein 7	S239	WVLVsPLsKEDAGEY	Secreted
Q8N6C5	IGSF1	Immunoglobulin superfamily member 1	S1329	SSTSQRIsVELPVPI	Secreted
Q14005	IL16	Pro-interleukin-16	S845	SIRQRIssFEtFGss	Secreted
Q14005	IL16	Pro-interleukin-16	S584	PPLRLKKsFEILVRK	Secreted
Q14005	IL16	Pro-interleukin-16	S471	GKERHQWsLEGVKRL	Secreted
Q14005	IL16	Pro-interleukin-16	S974	FPLtRsQsCETkLLD	Secreted
Q14005	IL16	Pro-interleukin-16	S614	SDSDPQKsLEERENS	Secreted
Q14005	IL16	Pro-interleukin-16	S1247	MSAGLGFsLEGGKGs	Secreted
P01583	IL1A	Interleukin-1 alpha	S104	LEAIANDsEEEIIKP	Secreted
Q17R60	IMPG1	Interphotoreceptor matrix proteoglycan 1	S312	TAIFKRHsAEAKsPA	Secreted
Q14641	INSL4	Early placenta insulin-like peptide	S90	SEFIPNLsPELKKPL	Secreted
P19827	ITIH1	Inter-alpha-trypsin inhibitor heavy chain H1	S129	QYRKAAIsGENAGLV	Secreted
P19823	ITIH2	Inter-alpha-trypsin inhibitor heavy chain H2	S60	QRsLPGEsEEMMEEV	Secreted
Q14624	ITIH4	Inter-alpha-trypsin inhibitor heavy chain H4	S225	TLSQQQKsPEQQETV	Secreted
P01042	KNG1	Kininogen-1	S332	EttCsKEsNEELtES	Secreted
P01042	KNG1	Kininogen-1	S329	VAREttCsKEsNEEL	Secreted
P01042	KNG1	Kininogen-1	S275	PRDIPTNsPELEETL	Secreted
P01042-2	KNG1	Isoform LMW of Kininogen-1	S406	ETtsHLRsCEYKGRP	Secreted
Q86UP2	KTN1	Kinectin	S1313	VIENsDVsPEtESSE	ER-m
Q86UP2	KTN1	Kinectin	S812	EKDGKIksVEELLEA	ER-m
Q86UP2	KTN1	Kinectin	S1289	DLHkAQQsLELIQSk	ER-m
P07942	LAMB1	Laminin subunit beta-1	S1666	KRKAAQNsGEAEyIE	Secreted
P07942	LAMB1	Laminin subunit beta-1	S1222	PYRETVDsVERKVSE	Secreted
P07942	LAMB1	Laminin subunit beta-1	S1478	RADEAkQsAEDILLK	Secreted
P07942	LAMB1	Laminin subunit beta-1	S1520	QDSADLDsIEAVANE	Secreted
P55268	LAMB2	Laminin subunit beta-2	S1548	LELSIPAsAEQIQHL	Secreted
P11047	LAMC1	Laminin subunit gamma-1	S1149	ERLIEIAsRELEKAK	Secreted
Q13753	LAMC2	Laminin subunit gamma-2	S686	LQLAKVRsQENSYQS	Secreted
P31025	LCN1	Lipocalin-1	S24	QAHHLLAsDEEIQDV	Secreted
P30533	LRPAP1	Alpha-2-macroglobulin receptor-associated protein	S50	KPsPKREsGEEFRME	Golgi/ER-l
P30533	LRPAP1	Alpha-2-macroglobulin receptor-associated protein	S247	RVsHQGYstEAEFEE	Golgi/ER-l
P30533	LRPAP1	Alpha-2-macroglobulin receptor-associated protein	S165	AKTSGKFsGEELDKL	Golgi/ER-l
Q14766	LTBP1	Latent-transforming growth factor beta-binding protein 1	S321	FPAQkGIsGEQstEG	Secreted
Q14766	LTBP1	Latent-transforming growth factor beta-binding protein 1	S1616	IQDRFLNsFEELQAE	Secreted

Q14766-3	LTBP1	Isoform 3 of Latent-transforming growth factor beta-binding protein 1	S408	PVEALTFsREHGPGV	Secreted
Q14766	LTBP1	Latent-transforming growth factor beta-binding protein 1	S325	kGIsGEQstEGSFPL	Secreted
Q14766	LTBP1	Latent-transforming growth factor beta-binding protein 1	S1414	FVPAGEsssEAGGEN	Secreted
Q14766-3	LTBP1 iso3	Isoform 3 of Latent-transforming growth factor beta-binding protein 1	S408	PVEALTFsREHGPGV	Secreted
Q14767	LTBP2	Latent-transforming growth factor beta-binding protein 2	S372	ARGHCANsCERGDTT	Secreted
Q14767	LTBP2	Latent-transforming growth factor beta-binding protein 2	S506	EEALVENsVETRPPP	Secreted
Q9NS15	LTBP3	Latent-transforming growth factor beta-binding protein 3	S1213	KPPRDEDsSEEDSDE	Secreted
Q9NS15	LTBP3	Latent-transforming growth factor beta-binding protein 3	S218	PLGPGQIsAEVQAPP	Secreted
P02788	LTF	Lactotransferrin	S540	ENKCVPNsNERYyGy	Secreted
P51884	LUM	Lumican	S138	SVGPLPKsLEDLQLt	Secreted
Q7Z553	MAMDC1	MAM domain-containing glycosylphosphatidylinositol anchor protein 2	S506	TLRIVNVsREMSGMy	PM
P43121	MCAM	Cell surface glycoprotein MUC18	S123	CQGKRPRsQEyRIQL	PM
P43121	MCAM	Cell surface glycoprotein MUC18	S238	SGNHMKEsREVTVPV	PM
Q8NFP4-2	MDGA1 iso2	Isoform 2 of MAM domain-containing glycosylphosphatidylinositol anchor protein 1	S948	CAWRGFLsVEGGCLG	PM
Q08431	MFGE8	Lactadherin	S42	GGLCEEIsQEVRGDV	Secreted
Q9UM21	MGAT4A	Alpha-1,3-mannosyl-glycoprotein 4-beta-N-acetylglucosaminyltransferase A	S474	KSEGLEIsKETKDKR	PM
Q9UM21	MGAT4A	Alpha-1,3-mannosyl-glycoprotein 4-beta-N-acetylglucosaminyltransferase A	S197	ANLEKEFsKEISSGL	PM
P08493	MGP	Matrix Gla protein	S22	VVTLCYEsHEsMEsY	Secreted
P08493	MGP	Matrix Gla protein	S25	LCYEsHEsMEsYELN	Secreted
P08493	MGP	Matrix Gla protein	S28	EsHEsMEsYELNPFI	Secreted
Q5JRA6	MIA3	Transport and Golgi organization protein 1 homolog	S727	VEEDDYPsEELLEDE	ER-m
Q5JRA6	MIA3	Transport and Golgi organization protein 1 homolog	S876	GEPEGELsKEDHENT	ER-m
Q5JRA6	MIA3	Transport and Golgi organization protein 1 homolog	S408	MDLEsSssEEEKEDD	ER-m
Q5JRA6	MIA3	Transport and Golgi organization protein 1 homolog	S407	TMDLEsSssEEEKED	ER-m
Q5JRA6	MIA3	Transport and Golgi organization protein 1 homolog	S1067	PLHEDNFsREKTAEL	ER-m
Q5JRA6	MIA3	Transport and Golgi organization protein 1 homolog	S186	PEPVEANsEESDSVF	ER-m
Q5JRA6	MIA3	Transport and Golgi organization protein 1 homolog	S358	PTDKEQNsNEEDKVQ	ER-m
P51511	MMP15	Matrix metalloproteinase-15	S589	GAEPGADsAEGDVGD	PM
Q9BU76	MMTAG2	Multiple myeloma tumor-associated protein 2	S220	EAtssPtsPERPRHH	PM
Q13421	MSLN	Mesothelin	S200	PGRFVAEsAEVLLPR	PM
P98088	MUC5AC	Mucin-5AC	S5628	APGDTQHsEEAEPEP	Secreted
P98088	MUC5AC	Mucin-5AC	S5643	SQEAESGsWERGVPV	Secreted

Q9NR99	MXRA5	Matrix-remodeling-associated protein 5	S1082	GDPTHsRssESEGQE	Secreted
Q9NR99	MXRA5	Matrix-remodeling-associated protein 5	S1525	ISQASRDsKENVFLN	Secreted
Q9NR99	MXRA5	Matrix-remodeling-associated protein 5	S291	LRQNRSRsIEEEQEQ	Secreted
Q9BRK3	MXRA8	Matrix remodeling-associated protein 8	S228	RLLDLYAsGERRAyG	PM
Q99972	MYOC	Myocilin	S199	ARAVPPGsREVsTWN	Secreted
P14543	NID1	Nidogen-1	S1181	VALDLAIsKEtDAFQ	Secreted
P01303	NPY	Pro-neuropeptide Y	S82	sDLLMREstENVPRT	Secreted
O60462	NRP2	Neuropilin-2	S781	GVIGKGRsGEIAIDD	PM
Q02818	NUCB1	Nucleobindin-1	S369	NAKAQRLsQEtEALG	ER/Golgi-l
Q02818	NUCB1	Nucleobindin-1	S86	DIksGKLsRELDFVs	ER/Golgi-l
Q9NPH6	OBP2B	Odorant-binding protein 2b	S153	LVQRKGLsEEDIFTP	Secreted
Q96PB7	OLFM3	Noelin-3	S99	KVQNMSQsIEVLNLR	Secreted
Q68BL7	OLFML2A	Olfactomedin-like protein 2A	S334	SNSAEPNsAEQDEAE	Secreted
Q68BL8	OLFML2B	Olfactomedin-like protein 2B	S258	TVSQQINsIELLQTR	Secreted
P78380	OLR1	Oxidized low-density lipoprotein receptor 1	S95	RQQAEEAsQEsENEL	Secreted
Q99983	OMD	Osteomodulin	S402	DPDNAHEsPEQEGAE	Secreted
Q13438	OS9	Protein OS-9	S509	KRLEEkQsPELVkKH	ER-l
Q32P28	P3H1	Prolyl 3-hydroxylase 1	S706	SPEEMDLsQEQPLDA	ER-l
Q32P28	P3H1	Prolyl 3-hydroxylase 1	S418	RETAVRIsQEIGNLM	ER-l
O15460	P4HA2	Prolyl 4-hydroxylase subunit alpha-2	S346	VRYYDVMsDEEIERI	ER-l
P07237	P4HB	Protein disulfide-isomerase	S331	MTkykPEsEELtAEr	ER-l
P07237	P4HB	Protein disulfide-isomerase	S357	KIKPHLMsQELPEDW	ER-l
P19021	PAM	Peptidyl-glycine alpha-amidating monooxygenase	S942	RKGFDRLstEGsDQE	Secreted
P19021	PAM	Peptidyl-glycine alpha-amidating monooxygenase	S957	KEDDGsEsEEEYSAP	Secreted
Q96QU1	PCDH15	Protocadherin-15	S858	NVSYRIRsPEVKHFF	PM
Q8NBP7	PCSK9	Proprotein convertase subtilisin/kexin type 9	S47	ELVLALRsEEDGLAE	Secreted
P01127	PDGFB	Platelet-derived growth factor subunit B	S196	VtRSPGGsQEQRAKT	Secreted
P07237	PDIA1	Protein disulfide-isomerase	S331	MTkykPEsEELtAEr	ER-l
P07237	PDIA1	Protein disulfide-isomerase	S357	KIKPHLMsQELPEDW	ER-l
P13667	PDIA4	Protein disulfide-isomerase A4	S470	KDLGLsEsGEDVNAA	ER-l
P13667	PDIA4	Protein disulfide-isomerase A4	S287	IEQSGPPsKEILTLk	ER-l
P36955	PEDF	Pigment epithelium-derived factor	S227	KFDSRKTsLEDFYLD	Secreted
Q13018	PLA2R1	Secretory phospholipase A2 receptor	S1272	STVLDSMsFEAAHEF	PM
Q8TD55	PLEKHQ1	Pleckstrin homology domain-containing family O member 2	S273	LPDKLKVsWENPsPQ	Secreted
Q8TD55	PLEKHQ1	Pleckstrin homology domain-containing family O member 2	S439	GsEPAPVsAETLLSQ	Secreted
Q8TD55	PLEKHQ1	Pleckstrin homology domain-containing family O member 2	S468	MRDLGELsQEAPGLR	Secreted
P00747	PLG	Plasminogen	S45	KKQLGAGsIEECAAK	Secreted
P00747	PLG	Plasminogen	S189	EEECMHCsGENYDGK	Secreted
P00747	PLG	Plasminogen	S477	LPDVETPsEEDCMFG	Secreted
P00747	PLG	Plasminogen	S358	SCDSSPVsTEQLAPT	Secreted
Q9NZ53	PODXL2	Podocalyxin-like protein 2	S195	ELLPVNGsQEEAKPQ	PM
Q7Z4H8	POGLUT3	Protein O-glucosyltransferase 3	S218	YTDFKMFsDEILLSL	ER-l
P01189	POMC	Pro-opiomelanocortin	S168	PNGAEDEsAEAFPLE	Secreted
P04280	PRB1	Basic salivary proline-rich protein 1	S24	QNLNEDVsQEESPSL	Secreted
P51888	PRELP	Prolargin	S322	HLYLNNNsIEKINGT	Secreted

Q92954	PRG4	Proteoglycan 4	S77	CKGRCFEsFERGREC	Secreted
P02810	PRH1	Salivary acidic proline-rich phosphoprotein 1/2	S24	QDLDEDVsQEDVPLV	Secreted
P14314	PRKCSH	Glucosidase 2 subunit beta	S168	ELQAGkksLEDQVEM	ER-l
Q9BZD6	PRRG4	Transmembrane gamma-carboxyglutamic acid protein 4	S38	AGEEVFTsKEEANFF	PM
Q16647	PTGIS	Prostacyclin synthase	S118	QLPHYSPsDEKARMK	PM
P01270	PTH	Parathyroid hormone	S48	NLGKHLNsMERVEWL	Secreted
Q92626	PXDN	Peroxidasin homolog	S1337	YHFRGRRsLEFSYQE	Secreted
O00391	QSOX1	Sulfhydryl oxidase 1	S426	ARQNVDHsQEAAKAK	Golgi-m
Q6ZRP7	QSOX2	Sulfhydryl oxidase 2	S578	YSADQGDssEGGTLA	Secreted
Q15293	RCN1	Reticulocalbin-1	S80	DQLtPDEskErLGkI	ER-l
Q15293	RCN1	Reticulocalbin-1	S234	EYIADMFsHEENGPE	ER-l
Q15293	RCN1	Reticulocalbin-1	S136	RDKDDkIsWEEYkQA	ER-l
Q14257	RCN2	Reticulocalbin-2	S207	KNGDGFVsLEEFLGD	ER-l
O95980	RECK	Reversion-inducing cysteine-rich protein with Kazal motifs	S620	RLCLSEHsSEDDRRT	PM
Q9BXY4	RSPO3	R-spondin-3	S237	EAIPDsKsLEssKEI	Secreted
Q9BXY4	RSPO3	R-spondin-3	S241	DsKsLEssKEIPEQR	Secreted
P13521	SCG2	Secretogranin-2	S268	TQEEVRDsKENIEKN	Secreted
P13521	SCG2	Secretogranin-2	S556	EHLNQGSsQETDKLA	Secreted
P13521	SCG2	Secretogranin-2	S532	KRVPGQGssEDDLQE	Secreted
P13521	SCG2	Secretogranin-2	S432	EALPDGLsVEDILNL	Secreted
P13521	SCG2	Secretogranin-2	S378	TGEKPNGsVEPEREL	Secreted
Q8WXD2	SCG3	Secretogranin-3	S37	SLHNRELsAERPLNE	Secreted
Q8WXD2	SCG3	Secretogranin-3	S362	FPAPsEKsHEETDST	Secreted
P05408	SCG5	Neuroendocrine protein 7B2	S205	KKSVPHFsDEDKDPE	Secreted
O60613	SELENOF	Selenoprotein F	S153	ILKWNTDsVEEFLSE	ER-l
Q9UK55	SERPINA10	Protein Z-dependent protease inhibitor	S56	EEDEQEAsEEKAsEE	Secreted
Q9UK55	SERPINA10	Protein Z-dependent protease inhibitor	S61	EAsEEKAsEEEKAWL	Secreted
P01011	SERPINA3	Alpha-1-antichymotrypsin	S302	tLKRWRDsLEFREIG	Secreted
P36955	SERPINF1	Pigment epithelium-derived factor	S227	KFDSRKTsLEDFYLD	Secreted
P50454	SERPINH1	Serpin H1	S98	SQAkAVLsAEQLRDE	ER-l
Q96LC7	SIGLEC10	Sialic acid-binding Ig-like lectin 10	S630	PLPPGAPsPESKKNQ	PM
Q9H173	SIL1	Nucleotide exchange factor SIL1	S147	EGAEMESsKEDKARQ	ER-l
Q9NY59	SMPD3	Sphingomyelin phosphodiesterase 3	S209	GsIKRTAsVEyKGDG	Golgi-m
P08294	SOD3	Extracellular superoxide dismutase [Cu-Zn]	S195	GRGGNQAsVENGNAG	Secreted
Q14515	SPARCL1	SPARC-like protein 1	S84	KEESHEQsAEQGKsS	Secreted
Q14515	SPARCL1	SPARC-like protein 1	S92	AEQGKsSsQELGLKD	Secreted
Q14515	SPARCL1	SPARC-like protein 1	S295	IQETEWQsQEGKTGL	Secreted
Q14515	SPARCL1	SPARC-like protein 1	S414	AKKAENSsNEEEtss	Secreted
Q14515	SPARCL1	SPARC-like protein 1	S420	SsNEEEtssEGNMRV	Secreted
Q14515	SPARCL1	SPARC-like protein 1	S59	NEKETAVsTEDDSHH	Secreted
Q92563	SPOCK2	Testican-2	S72	VEDDYIKsWEDNQQG	Secreted
Q9HCB6	SPON1	Spondin-1	S276	EEEIRQQsDEVLTVI	Secreted
P10451	SPP1	Osteopontin	S303	KHLKFRIsHELDsAs	Secreted
P10451	SPP1	Osteopontin	S63	APQNAVssEEtNDFK	Secreted
P10451	SPP1	Osteopontin	S62	LAPQNAVssEEtNDF	Secreted
P10451	SPP1	Osteopontin	S254	KRKANDEsNEHsDVI	Secreted
P10451	SPP1	Osteopontin	S234	tsQLDDQsAEtHsHK	Secreted
P10451	SPP1	Osteopontin	S224	WDsRGKDsyEtsQLD	Secreted
P10451	SPP1	Osteopontin	S263	EHsDVIDsQELsKVs	Secreted
P10451	SPP1	Osteopontin	S195	DItsHMEsEELNGAy	Secreted
P10451	SPP1	Osteopontin	S27	KQADsGssEEKQLYN	Secreted
P10451	SPP1	Osteopontin	S270	sQELsKVsREFHsHE	Secreted
P10451	SPP1	Osteopontin	S291	MLVVDPKsKEEDKHL	Secreted

P10451	SPP1	Osteopontin	S26	VKQADsGssEEKQLY	Secreted
P10451	SPP1	Osteopontin	S280	FHsHEFHsHEDMLVV	Secreted
P10451	SPP1	Osteopontin	S275	KVsREFHsHEFHsHE	Secreted
P10451	SPP1	Osteopontin	S120	DTDDsHQsDEsHHsD	Secreted
P10451	SPP1	Osteopontin	S126	QsDEsHHsDEsDELV	Secreted
P10451	SPP1	Osteopontin	S129	EsHHsDEsDELVtDF	Secreted
P10451	SPP1	Osteopontin	S78	QETLPsKsNEsHDHM	Secreted
Q13103	SPP2	Secreted phosphoprotein 24	S170	NYLFGLIsDESISEQ	Secreted
Q13103	SPP2	Secreted phosphoprotein 24	S96	ETTCRKDsGEDPATC	Secreted
A2VEC9	SSPO	SCO-spondin	S2421	REDCLDGsDERHCAR	Secreted
P02808	STATH	Statherin	S21	VsMIGADssEEKFLR	Secreted
P02808	STATH	Statherin	S22	sMIGADssEEKFLRR	Secreted
O76061	STC2	Stanniocalcin-2	S287	GAQGPSGssEWEDEQ	Secreted
Q8IWU5	SULF2	Extracellular sulfatase Sulf-2	S838	LGLkDGGsYEQYRQF	Secreted
Q2MV58-6	TCTN1	Isoform 6 of Tectonic-1	S201	SSPVSARsTEGEEPA	PM
P02787	TF	Serotransferrin	S298	EHFGKDKsKEFQLFs	Secreted
P02787	TF	Serotransferrin	S389	VGKIECVsAETTEDC	Secreted
P10646	TFPI	Tissue factor pathway inhibitor	S30	PAPLNADsEEDEEHT	Secreted
P01266	TG	Thyroglobulin	S2737	DGAKGGQsAEsEEEE	Secreted
P01266	TG	Thyroglobulin	S2740	KGGQsAEsEEEELTA	Secreted
O43493	TGOLN2	Trans-Golgi network integral membrane protein 2	S298	PHAFKtEsGEEtDLI	Golgi-m
O43493	TGOLN2	Trans-Golgi network integral membrane protein 2	S71	KDsPsKssAEAQtPE	Golgi-m
O43493	TGOLN2	Trans-Golgi network integral membrane protein 2	S351	EKMsGsAsSENREGT	Golgi-m
P35442	THBS2	Thrombospondin-2	S258	ttEYVGPssERRPEV	Secreted
Q9NS62	THSD1	Thrombospondin type-1 domain-containing protein 1	S463	SPSFRKNsDEENICE	Secreted
Q24JP5	TMEM132A	Transmembrane protein 132A	S529	AEPAAEAsDEAERRA	PM
Q9BTV4	TMEM43	Transmembrane protein 43	S292	LLHHGDFsAEEVFHR	Perinuc-m
P24821	TNC	Tenascin	S72	sVDLEsAsGEKDLAP	Secreted
P24821	TNC	Tenascin	S257	EICPVPCsEEHGTCV	Secreted
P24821	TNC	Tenascin	S616	CICNEGYsGEDCSEV	Secreted
P24821	TNC	Tenascin	S1310	TVPGSLRsMEIPGLR	Secreted
Q03169	TNFAIP2	Tumor necrosis factor alpha-induced protein 2	S114	AAAAGGVsEEELVRR	Secreted
P22105	TNXB	Tenascin-X	S3651	LAPAGQTsEEsRPRL	Secreted
P22105	TNXB	Tenascin-X	S581	CVCEDGYsGEDCGVR	Secreted
Q9NP99	TREM1	Triggering receptor expressed on myeloid cells 1	S143	GFSGtPGsNENSTQN	PM
P20396	TRH	Pro-thyrotropin-releasing hormone	S166	ADPKAQRsWEEEEEE	Secreted
Q6UY14	TSRC1	ADAMTS-like protein 4	S696	FLCISREsGEELDER	Secreted
P02766	TTR	Transthyretin	S72	ASGKTSEsGELHGLT	Secreted
Q8NBS9	TXNDC5	Thioredoxin domain-containing protein 5	S108	DLGDKYNsMEDAkVy	ER-l
Q76M96	URB	Coiled-coil domain-containing protein 80	S503	KAQDKILsNEyEEKY	Secreted
P19320	VCAM1	Vascular cell adhesion protein 1	S465	MKSLENKsLEMTFIP	PM
P13611	VCAN	Versican core protein	S1351	GHPIDsEsKEDEPCS	Secreted
P13611	VCAN	Versican core protein	S2116	EIESETtsEEQIQEE	Secreted
P13611	VCAN	Versican core protein	S2941	NKTDGQVsGEAIKMF	Secreted
P13611	VCAN	Versican core protein	S3209	AHLtsILsHEEQMFV	Secreted
O15240	VGF	Neurosecretory protein VGF	S420	AGAEDKRsQEEtPGH	Secreted
P04004	VTN	Vitronectin	S406	ATWLSLFssEESNLG	Secreted
P04004	VTN	Vitronectin	S407	TWLSLFssEESNLGA	Secreted
P04004	VTN	Vitronectin	S312	FAMMQRDsWEDIFEL	Secreted

Q6PCB0	VWA1	von Willebrand factor A domain-containing protein 1	S93	FPFGQHSsGEAAQDA	Secreted
P04275	VWF	von Willebrand factor	S1517	GEADFNRsKEFMEEV	Secreted
P04275	VWF	von Willebrand factor	S1613	MVTGNPAsDEIKRLP	Secreted
Q86Y38	XYLT1	Xylosyltransferase 1	S317	NVQWDEDsVEYMPAN	Golgi/ER-l

Table S3.

Acc. Numb.	Gene name	Protein name	Cell Comp.	Ref.
P02765	AHSG	Alpha-2-HS-glycoprotein	Secreted	[2,3]
P02647	APOA1	Apolipoprotein A-I	Secreted	[2]
P02652	APOA2	Apolipoprotein A-II	Secreted	[2]
P02649	APOE	Apolipoprotein E	Secreted	[2]
O14791	APOL1	Apolipoprotein L1	Secreted	[2]
O95972	BMP15	Bone morphogenetic protein 15	Secreted	[4]
P27797	CALR	Calreticulin	ER-l	[5]
O43852	CALU	Calumenin	ER-l	[2,5]
P05060	CHGB	Secretogranin 1	Secreted	[6]
P02662	CSN1S1	Alpha-S1-casein	Secreted	[7]
P01034	CST3	Cystatin-C	Secreted	[2]
O14958	CSQ2	Calsequestrin 2	ER-l	[5]
Q13316	DMP1	Dentin matrix acidic phosphoprotein 1	Secreted	[6,24]
Q96HE7	ERO1A	ERO1-like protein alpha	ER-l	[10]
P02671	FGA	Fibrinogen alpha chain	Secreted	[2]
Q9GZV9	FGF23	Fibroblast growth factor 23	Secreted	[12]
P02751	FN1	Fibronectin	Secreted	[2]
O60383	GDF9	Growth/differentiation factor 9	Secreted	[4]
P15515	HTN1	Histatin 1	Secreted	[13]
P23327	HRC	Sarcoplasmic reticulum histidine-rich calcium-binding protein	ER-l	[2,14]
P21815	IBSP	Bone sialoprotein 2	Secreted	[9]
P08833	IGFBP1	Insulin like growth factor binding protein 1	Secreted	[2]
P17936	IGFBP3	Insulin like growth factor binding protein 3	Secreted	[2]
P22692	IGFBP4	Insulin like growth factor binding protein 4	Secreted	[2]
P24593	IGFBP5	Insulin like growth factor binding protein 5	Secreted	[2]
Q16270	IGFBP7	Insulin like growth factor binding protein 7	Secreted	[2]
P05231	IL6	Interleukin-6	Secreted	[2]
Q9NQ76	MEPE	Matrix extracellular phosphoglycoprotein	Secreted	[6,24]
Q02818	NUCB1	Nucleobindin-1	ER-l	[2]
Q17RF5	ODAPH	Odontogenesis associated phosphoprotein	Secreted	[25]
P10451	SPP1	osteopontin	Secreted	[6,24,26]
Q16549	PCSK7	Proprotein convertase subtilisin/kexin type 7	Golgi-m	[17]
Q8NBP7	PCSK9	Proprotein convertase subtilisin/kexin type 9	Secreted	[2]
Q15063	POSTN	Periostin	Secreted	[27]
P07237	P4HB	Protein disulfide-isomerase	ER-l	[19]
P01210	PENK	Proenkephalin-A	Secreted	[2]
P05142	Prh1	Proline-rich protein HaeIII subfamily 1	Secreted	[20]
O00391	QSOX1	Sulfhydryl oxidase 1	Secreted	[2]
Q9BRK5	SDF4	45 kDa calcium-binding protein	Golgi-l	[22]
P13521	SCG2	Secretogranin 2	Secreted	[2]
Q8WXD2	SCG3	Secretogranin 3	Secreted	[2]
P05408	SCG5	Neuroendocrine protein 7B2	Secreted	[21]
Q13103	SPP2	Secreted phosphoprotein 24	Secreted	[2]
Q86TD4	SRL	Sarcalumenin	ER-l	[5]
P02808	STATH	Statherin	Secreted	[28]
O76061	STC2	Stanniocalcin 2	Secreted	[2]
Q13586	STIM1	Stromal interaction molecule 1	ER-l	[5]
Q13061	TRDN	Triadin	ER-l	[5]
P04275	VWF	von Willebrand factor	Secreted	[23]

Table S4.

Acc. Numb.	Gene name	Protein name	cells	pSite	Sequence	Cell Comp.
P02771	AFP	Alpha-fetoprotein	HepG2	s117	HSDCCSQsEEGRHNC	Secreted
P02771	AFP	Alpha-fetoprotein	HepG2	s344	RDFNQFSsGEKNIFL	Secreted
P02771	AFP	Alpha-fetoprotein	HepG2	s444	KKAPQLTsSELMAIT	Secreted
P02765	AHSG	Alpha-2-HS-glycoprotein	HepG2	s138	KCDSSPDsAEDVRKV	Secreted
P02765	AHSG	Alpha-2-HS-glycoprotein	HepG2	s330	VVSLGSPsGEVSHPR	Secreted
P02768	ALB	Albumin	HepG2	s82	KTCVADEsAENCDKS	Secreted
Q06481	APLP2	Amyloid beta precursor like protein 2	MDA	s590	PVDVRVSsEESEEIP	PM
P02652	APOA2	Apolipoprotein A-II	HepG2	s54	DLMEKVKsPELQAEA	Secreted
P02652	APOA2	Apolipoprotein A-II	HepG2	s68	AKSYFEKsKEQLTPL	Secreted
P04114	APOB	Apolipoprotein B-100	HepG2	s4048	TELRVREsDEETQIK	Secreted
P02649	APOE	Apolipoprotein E	HepG2	s147	VQAMLGQsTEELRVR	Secreted
O14791	APOL1	Apolipoprotein L1	HepG2	s311	PRVTEPIsAESGEQV	Secreted
O14791	APOL1	Apolipoprotein L1	HepG2	s314	TEPISAEsGEQVERV	Secreted
P05067	APP	Amyloid-beta precursor protein	HepG2	s441	HFQEKVEsLEQEAAN	PM
P12644	BMP4	Bone morphogenetic protein 4	HepG2/ MDA-MB-231	s91	RDLYRLQsGEEEEEQ	Secreted
Q8N4F0	BPIFB2	BPI fold-containing family B member 2	HepG2	s60	VPHFLDWsGEALQPT	Secreted
P01024	C3	Complement C3	HepG2	s38	PNILRLEsEETMVLE	Secreted
P01024	C3	Complement C3	HepG2	s70	PGKKLVLsSEKTVLT	Secreted
P01024	C3	Complement C3	HepG2	s297	RIPIEDGsGEVVLSR	Secreted
P01024	C3	Complement C3	HepG2	s1321	ESASLLRsEETKENE	Secreted
P01024	C3	Complement C3	HepG2	s1573	EQTIKSGsDEVQVGQ	Secreted
P0C0L4	C4A	Complement C4-A	HepG2	s918	LKVVARGsFEFPVGD	Secreted
O43852	CALU	Calumenin	HepG2/ MDA	s69	DQLTPEEsKERLGKI	Er/Golgi -l
P19022	CDH2	Cadherin-2	HepG2	s96	AVRSFPLsSEHAKFL	PM
P19022	CDH2	Cadherin-2	U2OS	s135	TEESVKEsAEVEEIV	PM
P05060	CHGB	Secretogranin-1	HepG2	s130	WAEGGGHsRERADEP	Secreted
P05060	CHGB	Secretogranin-1	HepG2	s225	ETHAAGHsQEKTHSR	Secreted
P05060	CHGB	Secretogranin-1	HepG2	s367	ERYRGRGsEEYRAPR	Secreted
P05060	CHGB	Secretogranin-1	HepG2	s377	YRAPRPQsEESWDEE	Secreted
Q9BU40	CHRDL1	Chordin-like protein 1	MDA	s185	CRGDGELsWEHSDGD	Secreted
Q07065	CKAP4	Cytoskeleton-associated protein 4	MDA	s232	ARERDFTsLENTVEE	ER-m/perinuc
P01034	CST3	Cystatin-C	HepG2/ MDA	s43	VGGPMDAsVEEEGVR	ER-m/perinuc
Q13217	DNAJC3	DnaJ homolog subfamily C member 3	HepG2	s274	KLNKLIEsAEELIRD	ER-l
Q9H8M9	EVA1A	Protein eva-1 homolog A	MDA	s114	LNKNVFTsAEELERA	PM
P12259	F5	Coagulation factor V	HepG2	s859	LGAGEFKsQEHAKHK	Secreted
P35555	FBN1	Fibrillin-1	MDA	s2702	GNPEPPVsGEMDDNS	Secreted
P02671	FGA	Fibrinogen alpha chain	HepG2	s56	DSDWPFCsDEDWNYK	Secreted
P02671	FGA	Fibrinogen alpha chain	HepG2	s357	TGTWNPGsSERGSAG	Secreted
P02679	FGG	Fibrinogen gamma chain	HepG2	s68	KVDKDLQsLEDILHQ	Secreted
P02751	FN1	Fibronectin	HepG2/ MDA	s2475	VQADREDsRE_____	Secreted
Q12841	FSTL1	Follistatin-related protein 1	MDA	s165	NGDSRLDsSEFLKFV	Secreted
O95633	FSTL3	Follistatin-related protein 3	MDA/ U2OS	s255	EEPPGGEsAEEEENF	Secreted

Q14393	GAS6	Growth arrest-specific protein 6	MDA	s71	ECVEELCsREEAREV	Secreted
Q8NBJ4	GOLM1	Golgi membrane protein 1	MDA	s309	VQAALSVsQENPEME	Golgi-m
P23327	HRC	Sarcoplasmic reticulum histidine-rich calcium-binding protein	HepG2	s119	KVGDEGVsGEEVFAE	ER-l
P23327	HRC	Sarcoplasmic reticulum histidine-rich calcium-binding protein	HepG2	s145	GSEDTEDsAEHRHHL	ER-l
P23327	HRC	Sarcoplasmic reticulum histidine-rich calcium-binding protein	HepG2	s358	EEEDEDVsTERWHQG	ER-l
P23327	HRC	Sarcoplasmic reticulum histidine-rich calcium-binding protein	HepG2	s431	REEDEEVsAELGHQA	ER-l
P23327	HRC	Sarcoplasmic reticulum histidine-rich calcium-binding protein	HepG2	s494	EKEEDPGsHEEDDES	ER-l
P23327	HRC	Sarcoplasmic reticulum histidine-rich calcium-binding protein	HepG2	s567	APLSPDHsEEEEEEE	ER-l
P08833	IGFBP1	Insulin-like growth factor-binding protein 1	HepG2	s194	LAKAQETsGEEISKF	Secreted
P08833	IGFBP1	Insulin-like growth factor-binding protein 1	HepG2	s242	NGKRIPGsPEIRGDP	Secreted
P17936	IGFBP3	Insulin-like growth factor-binding protein 3	HepG2	s148	EEDRSAGsVESPSVS	Secreted
P17936	IGFBP3	Insulin-like growth factor-binding protein 3	MDA	s201	STDTQNFsSESKRET	Secreted
P24593	IGFBP5	Insulin-like growth factor-binding protein 5	U2OS	s116	QVKIERDsREHEEPT	Secreted
Q16270	IGFBP7	Insulin-like growth factor-binding protein 7	MDA/ U2OS	s239	WVLVSPLsKEDAGEY	Secreted
P05231	IL6	Interleukin-6	MDA	s81	KSNMCESsKEALAEN	Secreted
P19823	ITIH2	Inter-alpha-trypsin inhibitor heavy chain H2	HepG2	s60	QRSLPGEsEEMMEEV	Secreted
P19823	ITIH2	Inter-alpha-trypsin inhibitor heavy chain H2	HepG2	s466	YDFLKRLsNENHGIA	Secreted
P19823	ITIH2	Inter-alpha-trypsin inhibitor heavy chain H2	HepG2	s886	DPEKPEAsMEVKGQK	Secreted
P01042	KNG1	Kininogen-1	HepG2	s332	ETTCSKEsNEELTES	Secreted
P07942	LAMB1	Laminin subunit beta-1	HepG2	s1682	VVYTVKQsAEDVKKT	Secreted
P07942	LAMB1	Laminin subunit beta-1	HepG2	1666	KRKAAQNsGEAEYIE	Secreted
P55268	LAMB2	Laminin subunit beta-2	HepG2	s1532	QEGADPDsIEMVATR	Secreted
P11047	LAMC1	Laminin subunit gamma-1	HepG2	s1149	ERLIEIAsRELEKAK	Secreted
Q14766	LTBP1	Latent-transforming growth factor beta-binding protein 1	MDA	s1414	FVPAGESsSEAGGEN	Secreted
O15232	MATN3	Matrilin-3	HepG2	s441	EEARRLVsTEDACGC	Secreted
Q08431	MFGE8	Lactadherin	MDA	s42	GGLCEEIsQEVRGDV	Secreted
Q9UM21	MGAT4A	Alpha-1,3-mannosyl-glycoprotein 4-beta-N-acetylglucosaminyltransferase A	HepG2	s474	KSEGLEIsKETKDKR	PM
Q5JRA6	MIA3	Transport and Golgi organization protein 1 homolog	HepG2	s226	HAQGEQAsFESFEEM	ER-m
Q5JRA6	MIA3	Transport and Golgi organization protein 1 homolog	HepG2	s229	GEQASFEsFEEMLQD	ER-m

Q13421	MSLN	Mesothelin	MDA/ U2OS	s200	PGRFVAEsAEVLLPR	PM
Q9BRK3	MXRA8	Matrix remodeling-associated protein 8	MDA	s228	RLLDLYAsGERRAYG	PM
Q02818	NUCB1	Nucleobindin-1	HepG2	s86	DIKSGKLsRELDFVS	ER/Golgi-l
Q02818	NUCB1	Nucleobindin-1	HepG2/ MDA	s369	NAKAQRLsQETEALG	ER/Golgi-l
P07237	P4HB	Protein disulfide-isomerase	HepG2	s357	KIKPHLMsQELPEDW	ER-l
Q8NBP7	PCSK9	Proprotein convertase subtilisin/kexin type 9	HepG2	s47	ELVLALRsEEDGLAE	Secreted
Q8NBP7	PCSK9	Proprotein convertase subtilisin/kexin type 9	HepG2/ MDA	s688	SRHLAQAsQELQ___	Secreted
P14314	PRKCSH	Glucosidase 2 subunit beta	HepG2/ MDA	s168	ELQAGKKsLEDQVEM	ER-l
P04070	PROC	Vitamin K-dependent protein C	HepG2	s347	TGWGYHSsREKEAKR	Secreted
O00391	QSOX1	Sulfhydryl oxidase 1	HepG2	s426	ARQNVDHsQEAAKAK	Golgi-m
Q15293	RCN1	Reticulocalbin-1	HepG2/ MDA	s80	DQLTPDEsKERLGKI	ER-l
Q9UK55	SERPINA 10	Protein Z-dependent protease inhibitor	HepG2	s56	EEDEQEAsEEKASEE	Secreted
F8WFD9	SHISA5	Shisa family member 5	U2OS	s115	LGQQGGDsAEQAPRG	ER-m
Q92563	SPOCK2	Testican-2	HepG2	s72	VEDDYIKsWEDNQQG	Secreted
P10451	SPP	Osteopontin	HepG2	s234	TSQLDDQsAETHSHK	Secreted
P10451	SPP1	Osteopontin	HepG2	s26	VKQADSGsSEEKQLY	Secreted
P10451	SPP1	Osteopontin	HepG2	s27	KQADSGSsEEKQLYN	Secreted
P10451	SPP1	Osteopontin	HepG2	S62	LAPQNAVsSEETNDF	Secreted
P10451	SPP1	Osteopontin	HepG2	s63	APQNAVSsEETNDFK	Secreted
P10451	SPP1	Osteopontin	HepG2	s195	DITSHMEsEELNGAY	Secreted
P10451	SPP1	Osteopontin	HepG2	s224	WDSRGKDsYETSQLD	Secreted
P10451	SPP1	Osteopontin	HepG2	s254	KRKANDEsNEHSDVI	Secreted
P10451	SPP1	Osteopontin	HepG2	s263	EHSDVIDsQELSKVS	Secreted
P10451	SPP1	Osteopontin	HepG2	s270	SQELSKVsREFHSHE	Secreted
P10451	SPP1	Osteopontin	HepG2	s275	KVSREFHsHEFHSHE	Secreted
P10451	SPP1	Osteopontin	HepG2	s280	FHSHEFHsHEDMLVV	Secreted
P10451	SPP1	Osteopontin	HepG2	s291	MLVVDPKsKEEDKHL	Secreted
P10451	SPP1	Osteopontin	HepG2	s303	KHLKFRIsHELDSAS	Secreted
P10451	SPP1	Osteopontin	HepG2	s310	SHELDSAsSEVN___	Secreted
O76061	STC2	Stanniocalcin-2	HepG2	s251	HHLPEPSsRETGRGA	Secreted
P02787	TF	Serotransferrin	HepG2	s389	VGKIECVsAETTEDC	Secreted
O43493	TGOLN2	Trans-Golgi network integral membrane protein 2	HepG2	s71	KDSPSKSsAEAQTPE	Golgi-m
O43493	TGOLN2	Trans-Golgi network integral membrane protein 2	HepG2	s298	PHAFKTEsGEETDLI	Golgi-m
O43493	TGOLN2	Trans-Golgi network integral membrane protein 2	HepG2	s351	EKMSGSAsSENREGT	Golgi-m
Q24JP5	TMEM132 A	Transmembrane protein 132A	MDA	s529	AEPAAEAsDEAERRA	PM
P24821	TNC	Tenascin	MDA	s72	SVDLESAsGEKDLAP	Secreted
P13611	VCAN	Versican core protein	HepG2	s2116	EIESETTsEEQIQEE	Secreted
O15240	VGF	Neurosecretory protein VGF	HepG2	s420	AGAEDKRsQEETPGH	Secreted
Q6PCB0	VWA1	von Willebrand factor A domain-containing protein 1	HepG2	s93	FPFGQHSsGEAAQDA	Secreted

Supplementary References

1. Käll, L.; Krogh, A.; Sonnhammer, E.L.L. Advantages of Combined Transmembrane Topology and Signal Peptide Prediction--the Phobius Web Server. *Nucleic Acids Res* **2007**, *35*, W429-432, doi:10.1093/nar/gkm256.
2. Tagliabracci, V.S.; Wiley, S.E.; Guo, X.; Kinch, L.N.; Durrant, E.; Wen, J.; Xiao, J.; Cui, J.; Nguyen, K.B.; Engel, J.L.; et al. A Single Kinase Generates the Majority of the Secreted Phosphoproteome. *Cell* **2015**, *161*, 1619–1632, doi:10.1016/j.cell.2015.05.028.
3. Kovářová, M.; Kalbacher, H.; Peter, A.; Häring, H.-U.; Didangelos, T.; Stefan, N.; Birkenfeld, A.; Schleicher, E.; Kantartzis, K. Detection and Characterization of Phosphorylation, Glycosylation, and Fatty Acid Bound to Fetuin A in Human Blood. *J Clin Med* **2021**, *10*, 411, doi:10.3390/jcm10030411.
4. Tibaldi, E.; Arrigoni, G.; Martinez, H.M.; Inagaki, K.; Shimasaki, S.; Pinna, L.A. Golgi Apparatus Casein Kinase Phosphorylates Bioactive Ser-6 of Bone Morphogenetic Protein 15 and Growth and Differentiation Factor 9. *FEBS Lett* **2010**, *584*, 801–805, doi:10.1016/j.febslet.2009.12.052.
5. Pollak, A.J.; Liu, C.; Gudlur, A.; Mayfield, J.E.; Dalton, N.D.; Gu, Y.; Chen, J.; Heller Brown, J.; Hogan, P.G.; Wiley, S.E.; et al. A Secretory Pathway Kinase Regulates Sarcoplasmic Reticulum Ca2+ Homeostasis and Protects against Heart Failure. *Elife* **2018**, *7*, e41378, doi:10.7554/eLife.41378.
6. Tagliabracci, V.S.; Engel, J.L.; Wen, J.; Wiley, S.E.; Worby, C.A.; Kinch, L.N.; Xiao, J.; Grishin, N.V.; Dixon, J.E. Secreted Kinase Phosphorylates Extracellular Proteins That Regulate Biomineralization. *Science* **2012**, *336*, 1150–1153, doi:10.1126/science.1217817.
7. Mackinlay, A.G.; West, D.W.; Manson, W. Specific Casein Phosphorylation by a Casein Kinase from Lactating Bovine Mammary Gland. *Eur J Biochem* **1977**, *76*, 233–243, doi:10.1111/j.1432-1033.1977.tb11588.x.
8. Szymanski, E.S.; Farrell, H.M. Isolation and Solubilization of Casein Kinase from Golgi Apparatus of Bovine Mammary Gland and Phosphorylation of Peptides. *Biochim Biophys Acta* **1982**, *702*, 163–172, doi:10.1016/0167-4838(82)90498-8.
9. Lasa-Benito, M.; Marin, O.; Meggio, F.; Pinna, L.A. Golgi Apparatus Mammary Gland Casein Kinase: Monitoring by a Specific Peptide Substrate and Definition of Specificity Determinants. *FEBS Lett* **1996**, *382*, 149–152, doi:10.1016/0014-5793(96)00136-6.
10. Zhang, J.; Zhu, Q.; Wang, X.; Yu, J.; Chen, X.; Wang, J.; Wang, X.; Xiao, J.; Wang, C.-C.; Wang, L. Secretory Kinase Fam20C Tunes Endoplasmic Reticulum Redox State via Phosphorylation of Ero1α. *EMBO J* **2018**, *37*, e98699, doi:10.15252/embj.201798699.
11. Chen, X.; Zhang, J.; Liu, P.; Wei, Y.; Wang, X.; Xiao, J.; Wang, C.; Wang, L. Proteolytic Processing of Secretory Pathway Kinase Fam20C by Site-1 Protease Promotes Biomineralization. *Proceedings of the National Academy of Sciences* **2021**, *118*, e2100133118, doi:10.1073/pnas.2100133118.
12. Tagliabracci, V.S.; Engel, J.L.; Wiley, S.E.; Xiao, J.; Gonzalez, D.J.; Nidumanda Appaiah, H.; Koller, A.; Nizet, V.; White, K.E.; Dixon, J.E. Dynamic Regulation of FGF23 by Fam20C Phosphorylation, GalNAc-T3 Glycosylation, and Furin Proteolysis. *Proc Natl Acad Sci U S A* **2014**, *111*, 5520–5525, doi:10.1073/pnas.1402218111.
13. Lamkin, M.S.; Lindhe, P. Purification of Kinase Activity from Primate Parotid Glands. *J Dent Res* **2001**, *80*, 1890–1894, doi:10.1177/00220345010800100601.
14. Pollak, A.J.; Haghighi, K.; Kunduri, S.; Arvanitis, D.A.; Bidwell, P.A.; Liu, G.-S.; Singh, V.P.; Gonzalez, D.J.; Sanoudou, D.; Wiley, S.E.; et al. Phosphorylation of Serine96 of Histidine-Rich Calcium-Binding Protein by the Fam20C Kinase Functions to Prevent Cardiac Arrhythmia. *Proc Natl Acad Sci U S A* **2017**, *114*, 9098–9103, doi:10.1073/pnas.1706441114.

15. Christensen, B.; Schytte, G.N.; Scavenius, C.; Enghild, J.J.; McKee, M.D.; Sørensen, E.S. FAM20C-Mediated Phosphorylation of MEPE and Its Acidic Serine- and Aspartate-Rich Motif. *JBMR Plus* **2020**, *4*, e10378, doi:10.1002/jbm4.10378.
16. Schytte, G.N.; Christensen, B.; Bregenov, I.; Kjøge, K.; Scavenius, C.; Petersen, S.V.; Enghild, J.J.; Sørensen, E.S. FAM20C Phosphorylation of the RGDSVVYGLR Motif in Osteopontin Inhibits Interaction with the Avβ3 Integrin. *J Cell Biochem* **2020**, *121*, 4809–4818, doi:10.1002/jcb.29708.
17. Ashraf, Y.; Duval, S.; Sachan, V.; Essalmani, R.; Susan-Resiga, D.; Roubtsova, A.; Hamelin, J.; Gerhardy, S.; Kirchhofer, D.; Tagliabracci, V.S.; et al. Proprotein Convertase 7 (PCSK7) Reduces apoA-V Levels. *FEBS J* **2020**, *287*, 3565–3578, doi:10.1111/febs.15212.
18. Ben Djoudi Ouadda, A.; Gauthier, M.-S.; Susan-Resiga, D.; Girard, E.; Essalmani, R.; Black, M.; Marcinkiewicz, J.; Forget, D.; Hamelin, J.; Evagelidis, A.; et al. Ser-Phosphorylation of PCSK9 (Proprotein Convertase Subtilisin-Kexin 9) by Fam20C (Family With Sequence Similarity 20, Member C) Kinase Enhances Its Ability to Degrade the LDLR (Low-Density Lipoprotein Receptor). *Arterioscler Thromb Vasc Biol* **2019**, *39*, 1996–2013, doi:10.1161/ATVBAHA.119.313247.
19. Yu, J.; Li, T.; Liu, Y.; Wang, X.; Zhang, J.; Wang, X.; Shi, G.; Lou, J.; Wang, L.; Wang, C.-C.; et al. Phosphorylation Switches Protein Disulfide Isomerase Activity to Maintain Proteostasis and Attenuate ER Stress. *EMBO J* **2020**, *39*, e103841, doi:10.15252/embj.2019103841.
20. Brunati, A.M.; Marin, O.; Bisinella, A.; Salviati, A.; Pinna, L.A. Novel Consensus Sequence for the Golgi Apparatus Casein Kinase, Revealed Using Proline-Rich Protein-1 (PRP1)-Derived Peptide Substrates. *Biochem J* **2000**, *351 Pt 3*, 765–768.
21. Ramos-Molina, B.; Lindberg, I. Phosphorylation and Alternative Splicing of 7B2 Reduce Prohormone Convertase 2 Activation. *Mol Endocrinol* **2015**, *29*, 756–764, doi:10.1210/me.2014-1394.
22. Hecht, T.K.-H.; Blank, B.; Steger, M.; Lopez, V.; Beck, G.; Ramazanov, B.; Mann, M.; Tagliabracci, V.; von Blume, J. Fam20C Regulates Protein Secretion by Cab45 Phosphorylation. *J Cell Biol* **2020**, *219*, e201910089, doi:10.1083/jcb.201910089.
23. Da, Q.; Han, H.; Valladolid, C.; Fernández, M.; Khatlani, T.; Pradhan, S.; Nolasco, J.; Matsunami, R.K.; Engler, D.A.; Cruz, M.A.; et al. In Vitro Phosphorylation of von Willebrand Factor by FAM20c Enhances Its Ability to Support Platelet Adhesion. *J Thromb Haemost* **2019**, *17*, 866–877, doi:10.1111/jth.14426.
24. Ishikawa, H.O.; Xu, A.; Ogura, E.; Manning, G.; Irvine, K.D. The Raine Syndrome Protein FAM20C Is a Golgi Kinase That Phosphorylates Bio-Mineralization Proteins. *PLoS One* **2012**, *7*, e42988, doi:10.1371/journal.pone.0042988.
25. Govitvattana, N.; Kaku, M.; Ohyama, Y.; Jaha, H.; Lin, I.-P.; Mochida, H.; Pavasant, P.; Mochida, Y. Molecular Cloning of Mouse Homologue of Enamel Protein C4orf26 and Its Phosphorylation by FAM20C. *Calcif Tissue Int* **2021**, *109*, 445–454, doi:10.1007/s00223-021-00847-y.
26. Lasa, M.; Chang, P.L.; Prince, C.W.; Pinna, L.A. Phosphorylation of Osteopontin by Golgi Apparatus Casein Kinase. *Biochem Biophys Res Commun* **1997**, *240*, 602–605, doi:10.1006/bbrc.1997.7702.
27. Lin, J.-H.; Lin, I.-P.; Ohyama, Y.; Mochida, H.; Kudo, A.; Kaku, M.; Mochida, Y. FAM20C Directly Binds to and Phosphorylates Periostin. *Sci Rep* **2020**, *10*, 17155, doi:10.1038/s41598-020-74400-6.
28. Manconi, B.; Cabras, T.; Vitali, A.; Fanali, C.; Fiorita, A.; Inzitari, R.; Castagnola, M.; Messana, I.; Sanna, M.T. Expression, Purification, Phosphorylation and Characterization of Recombinant Human Statherin. *Protein Expr Purif* **2010**, *69*, 219–225, doi:10.1016/j.pep.2009.07.015.

Supplementary FIGURES

Figure S1. Pathview of the Complement and Coagulation Cascades

Pathway visualization using Pathview Web [53] highlighting:

(A) Validated FAM20C substrates only.

(B) Putative FAM20C substrates (from Table 2).

FAM20C validated and putative substrates are marked in red.

Figure S2. Comparative visualization of consensus tree and networks.

A. Consensus tree derived from multiple source trees and rooted with *Pleurodeles waltl*. Node values represent the percentage of support across the source trees. B. Consensus network, highlighting the conflicting phylogenetic signal in the reticulated zone, with thick lines, that includes the species Homo sapiens C. Supernetwork, which illustrates the radial arrangement of Homo sapiens. It is seen connected in many directions, implying that it acts like a hub.

A

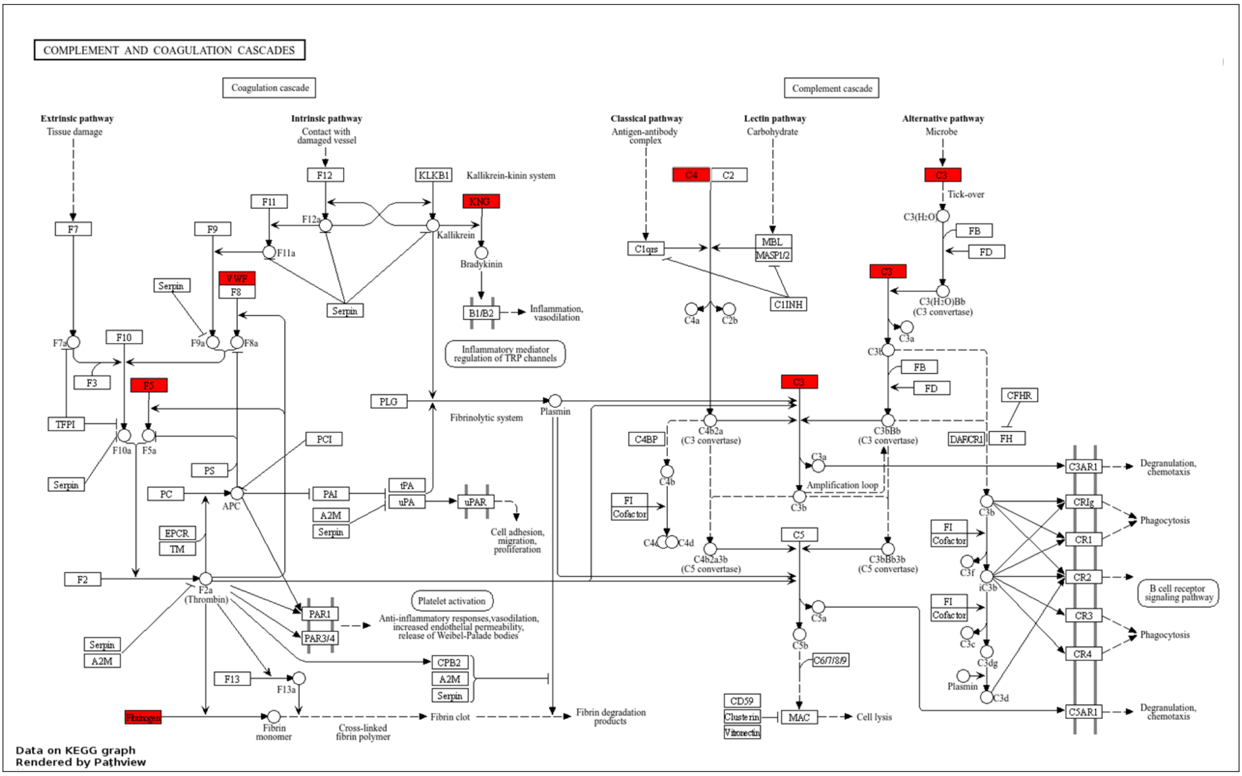
COMPLEMENT AND COAGULATION CASCADES
Coagulation cascade
Complement cascade
Extrinsic pathway
Tissue damage
Intrinsic pathway
Contact with damaged vessel
Classical pathway
Antigen-antibody complex
Lectin pathway
Carbohydrate
Alternative pathway
Microbe
Kallikrein-kinin system
Fibrinolytic system
Inflammation, vasodilation
Inflammatory mediator regulation of TRP channels
Cell adhesion, migration, proliferation
Platelet activation
Anti-inflammatory responses, vasodilation, increased endothelial permeability, release of Weibel-Palade bodies
Fibrin clot
Fibrin degradation products
Cross-linked fibrin polymer
Amplification loop
Cell lysis
Degranulation, chemotaxis
Phagocytosis
B cell receptor signaling pathway
Data on KEGG graph
Rendered by Pathview

B

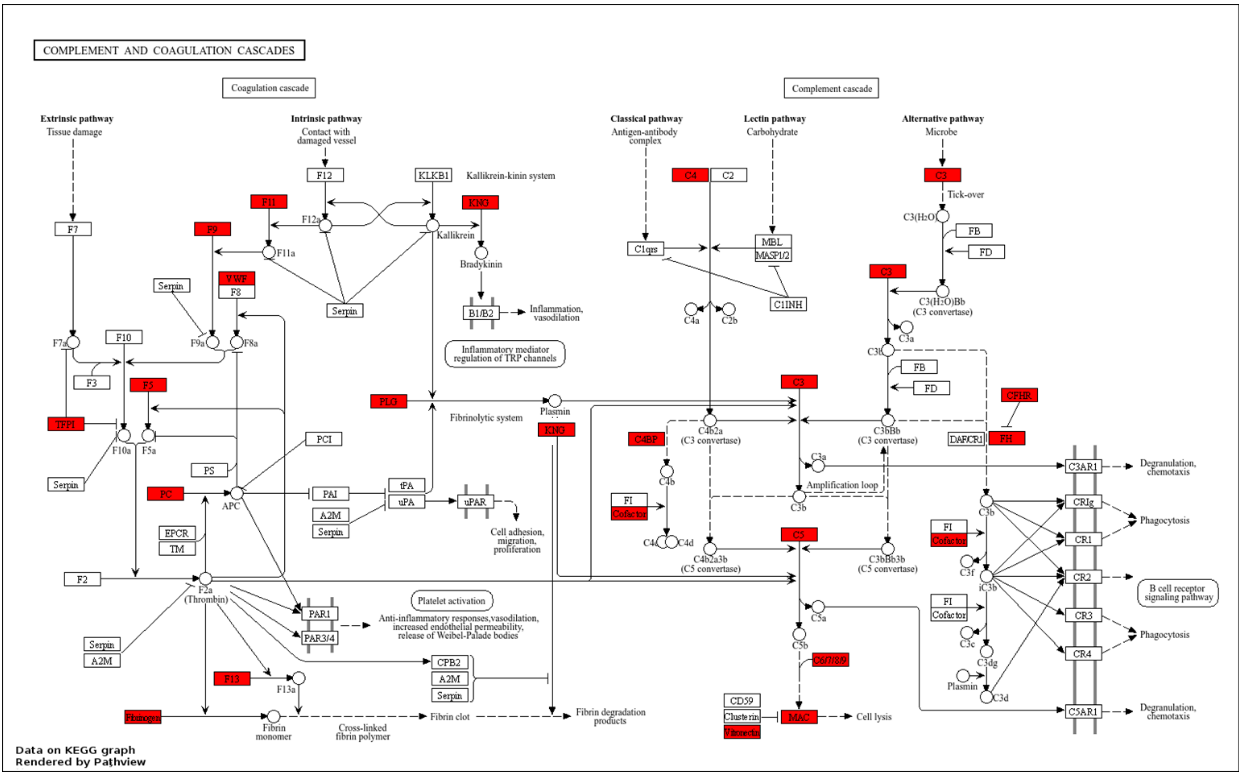
COMPLEMENT AND COAGULATION CASCADES
Coagulation cascade
Complement cascade
Extrinsic pathway
Tissue damage
Intrinsic pathway
Contact with damaged vessel
Classical pathway
Antigen-antibody complex
Lectin pathway
Carbohydrate
Alternative pathway
Microbe
Kallikrein-kinin system
Fibrinolytic system
Inflammation, vasodilation
Inflammatory mediator regulation of TRP channels
Cell adhesion, migration, proliferation
Platelet activation
Anti-inflammatory responses, vasodilation, increased endothelial permeability, release of Weibel-Palade bodies
Fibrin clot
Fibrin degradation products
Cross-linked fibrin polymer
Amplification loop
Cell lysis
Degranulation, chemotaxis
Phagocytosis
B cell receptor signaling pathway
Data on KEGG graph
Rendered by Pathview

Figure S1

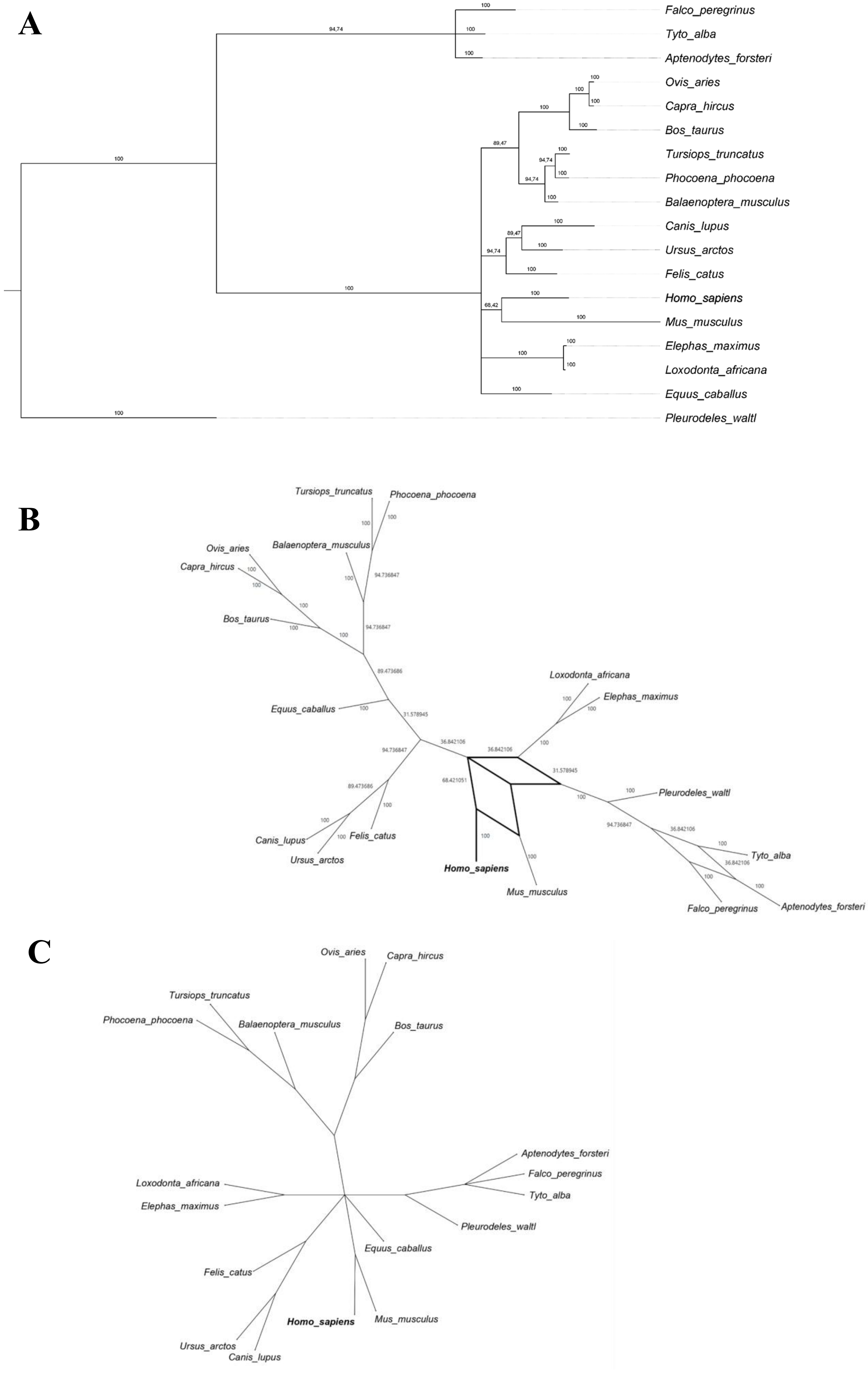

A
Falco_peregrinus
Tyto_alba
Aptenodytes_forsteri
Ovis_aries
Capra_hircus
Bos_taurus
Tursiops_truncatus
Phocoena_phocoena
Balaenoptera_musculus
Canis_lupus
Ursus_arctos
Felis_catus
Homo_sapiens
Mus_musculus
Elephas_maximus
Loxodonta_africana
Equus_caballus
Pleurodeles_waltl
B
Tursiops_truncatus
Phocoena_phocoena
Balaenoptera_musculus
Ovis_aries
Capra_hircus
Bos_taurus
Equus_caballus
Loxodonta_africana
Elephas_maximus
Pleurodeles_waltl
Canis_lupus
Ursus_arctos
Felis_catus
Homo_sapiens
Mus_musculus
Tyto_alba
Falco_peregrinus
Aptenodytes_forsteri
C
Ovis_aries
Capra_hircus
Tursiops_truncatus
Phocoena_phocoena
Balaenoptera_musculus
Bos_taurus
Aptenodytes_forsteri
Falco_peregrinus
Tyto_alba
Loxodonta_africana
Elephas_maximus
Pleurodeles_waltl
Equus_caballus
Felis_catus
Homo_sapiens
Mus_musculus
Ursus_arctos
Canis_lupus

Figure S2
